# Design, Synthesis, Biological Studies and Putative Binding Mode of Novel Benzotriazolothalidomide Derivatives as Cereblon Ligands

**DOI:** 10.1111/cbdd.70394

**Published:** 2026-09-14

**Authors:** Pramit Ganguly, Mrinalkanti Kundu, Tonmoy Sarkar, Gillian Dcosta, Kankana Banerjee, Ritama Banerjee, Arun K. Hajra, Sk Sahabuddin, Sohini Basu, Avratanu Das, Suparna Das, Rajib Barik, Md Ashif Ali

**Affiliations:** ^1^ TCG Lifesciences Pvt. Ltd. Kolkata India; ^2^ Department of Chemistry Ramakrishna Mission Residential College (Autonomous) Kolkata India

**Keywords:** benzotriazolo thalidomides, biological studies, cereblon E3 ligase, docking, ligand‐based design, synthesis

## Abstract

Targeted protein degradation (TPD) represents a new paradigm in drug discovery, encompassing several innovative strategies like proteolysis targeting chimeras (PROTACs). PROTACs work by linking the target protein of interest (POI) to an E3 ubiquitin ligase, marking it for ubiquitination, and subsequent degradation by the proteasome. The ligand space of E3 ligase cereblon (CRBN) mostly includes phthalimide‐based thalidomide 1 and its analogs, commonly known as cereblon E3 ligase modulators (CELMoDs) which have been approved for the treatment of multiple subtypes of non‐Hodgkin's lymphoma (NHL). Recently, benzotriazolo thalidomide (4) was reported which displayed similar binding mode like thalidomide, having relatively stronger binding affinity. Towards our aim of identifying new generation CRBN binding ligands, which can have use either in PROTAC or CELMoD chemical space, systematic design and SAR studies around 4, synthesis, biological, and docking experiments to understand putative binding mode have been carried out. As a result, a few compounds were identified as potent CRBN ligands, including compound 13 having an IC_50_ value of 198 ± 7 nM. Cell viability assay of benzotriazolothalidomide 13 in human embryonic kidney cells (HEK293) suggest a high level of safety profile (SI = > 500). To showcase the drug likeness of this compound, it was tested in preliminary ADME assays, revealing its excellent aqueous solubility (161.87 ± 0.96 μM), and high in vitro metabolic stability in human liver microsome (CL_int_, app = 16.57 ± 0.19 μL/min/mg of protein; T_1/2_ = 104.57 ± 1.22 min) and thus offers the scope for further optimization of the compound.

## Graphical abstract

We narrated the identification of benzotriazole‐based cereblon ligands for their potential use either in PROTAC or CELMoD chemical space, through systematic design and SAR studies, synthesis, biological, and docking experiments resulting in a non‐cytotoxic, potent, drug‐like compound 13, offering scope for optimization to yield an in vivo efficacious anti‐tumor agent.

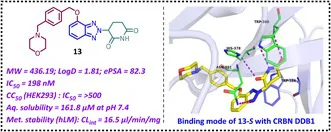

## 1 Introduction

Proteins play important roles within cellular systems and even if a single protein malfunctions, this can result in disease states. Consequently, identifying small molecular entities, which can modulate dysregulated proteins has been actively pursued in drug discovery research. Nearly 20 K proteins have been identified in human cells, but despite significant efforts made in research and development, still many proteins remain stubbornly undruggable. While some proteins, be they enzymes or receptors, lack an orthosteric binding site to either activate or inhibit or agonize or antagonize, many others are challenging to access, being present in the dense chemical “soup” of the nucleus and cytoplasm. Thus, traditional drug discovery efforts have predominantly concentrated on the druggable proteins as targets. Since the importance of many of these proteins is vital for cellular homeostasis, research focusing on difficult to target proteins may offer new avenues for treating currently intractable diseases (Paudel et al. 2023).

In that regard, targeted protein degradation (TPD) represents a powerful chemical biology approach that has profoundly impacted the recent field of drug discovery, enabling the degradation of difficult to target proteins and thus, is rapidly gaining momentum as an alternative to small‐molecule inhibitor (Zhao et al. 2022; Konstantinidou et al. 2019). A major TPD approach is the use of proteolysis targeting chimeras (PROTACs), which primarily utilize the ubiquitin‐proteasome system (UPS) pathways for selective protein degradation (Ahn et al. 2021; Chen et al. 2022; Guedeney et al. 2023; Zou et al. 2019). PROTACs consists of two recruiter moieties joined with a linker forming a ternary complex (Donoghue et al. 2020). One is a ligand of the protein of interest (POI, i.e., the one to be degraded) and the other a ligand of an E3 ligase. Productive ternary complexes result in poly‐Lys‐ubiquitination of the target protein leading to its shuttling to the 26S proteasome and subsequent degradation. On the other hand, molecular glues, which have smaller molecular weights and comply with Lipinski's rule of five and thus offer better drugability, mainly induces or stabilizes the protein–protein interaction (PPI) between ubiquitin‐ligase and substrate proteins, subsequently also leading to polyubiquitination and proteasome mediated degradation (Dewey et al. 2023; Sasso et al. 2022; Dong et al. 2021). Consequently, many PROTACs and molecular glues have progressed into clinical phase investigation and most recently, Veppanu (vepdegestrant, ARV‐471), an oral, targeted protein degrader has been approved by the FDA for treating adults with ER‐positive, HER2‐negative, ESR1‐mutated advanced, or metastatic breast cancer.

**FIGURE 1:**
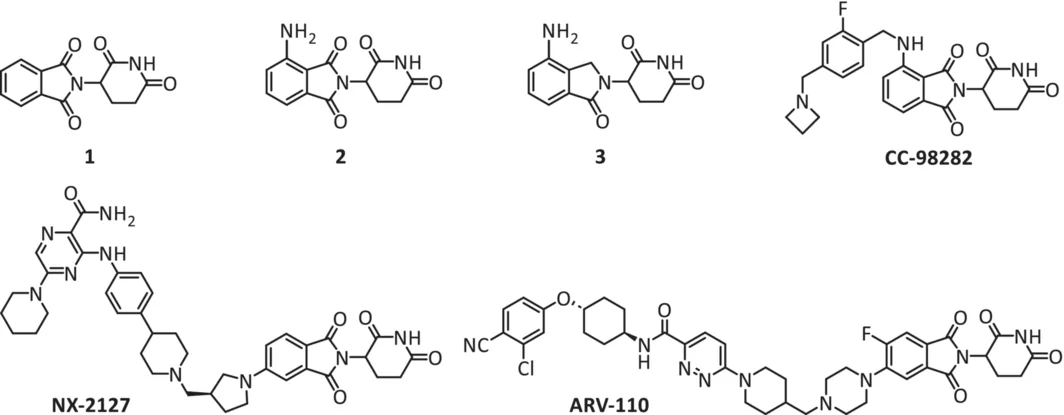
CRBN ligands and a few thalidomide‐containing clinical compounds.

The cullin‐damaged DNA‐binding‐RING box‐domain protein (CUL4‐DDB1‐RBX1‐CRBN) or simply CRL4^CRBN^ is one of the most important E3 ligases, that has been employed for both PROTAC and molecular glue development so far (Jones 2025; Girardini et al. 2019; Steinebach et al. 2019). The ligand space of CRBN mostly includes phthalimide‐based thalidomide (1) and its analogs pomalidomide (2) and lenalidomide (3) but a variety of ligands are also available in the literature (Xiao et al. 2020; Krasavin et al. 2023; Yan and Zhen 2023; Rodrigo‐Brenni et al. 2026; Ren et al. 2025). The therapeutic benefits of the treatments of these immunomodulatory drugs are connected to their ability to promote recruitment and ubiquitination of substrate proteins by CRL4^CRBN^ E3 ubiquitin ligase, with the resulting ubiquitin‐tagged proteins directed to and subsequently degraded by the 26S proteasome. A few PROTACs having phthalimide‐based core as in thalidomide, viz. Androgen receptor (AR) degrader ARV‐110, Bruton's tyrosine kinase (BTK) degrader NX‐2127, and molecular glue CC‐99282 targeting Ikaros family zinc finger protein 1/3 (IKZF1/IKZF3), are in various stages of ongoing clinical trials (Figure 1) (Cheng et al. 2025; Hollingsworth et al. 2023; Weiss et al. 2024).

**FIGURE 2:**
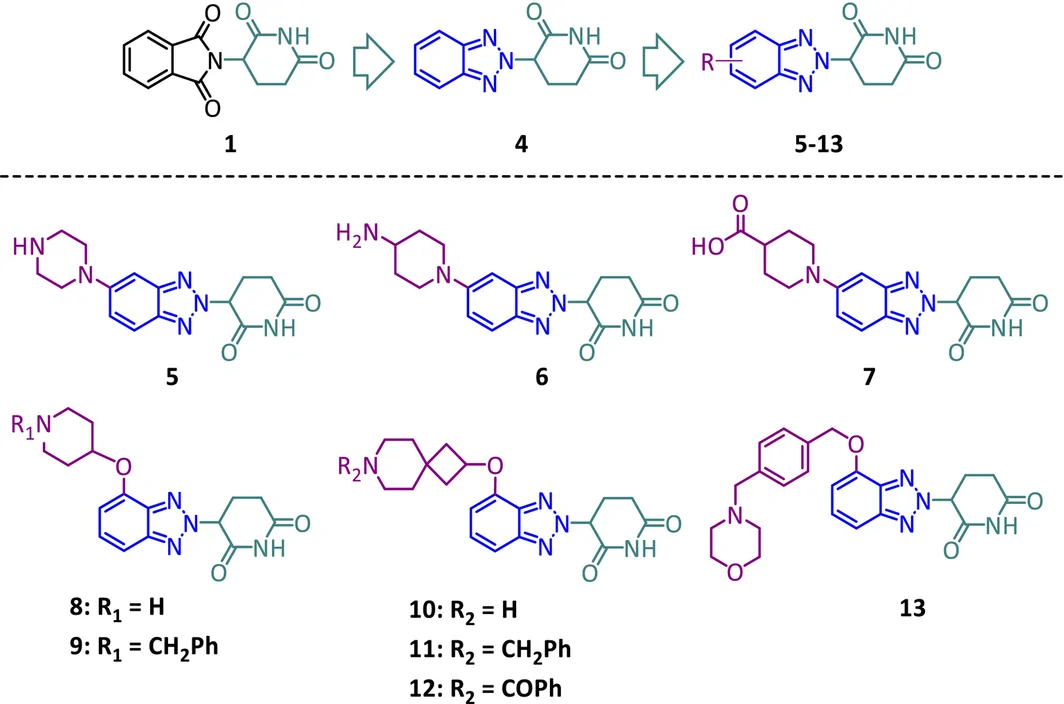
Design of new CRBN ligands.

Structural requirements for CRBN ligand binding have recently been elucidated (Boichenko et al. 2018; Heim et al. 2019) revealing that the binding mode of thalidomide (1) includes a hydrogen bond between one of the phthalimide carbonyl groups and a conserved asparagine residue of the protein, which contributes to the affinity between the two molecules. However, the other phthalimide carbonyl is not found to be involved in any specific interactions, not even with water molecules (Hartmann et al. 2015). In search of alternatives to the phthalimide‐glutarimide ligand, it was (Krasavin et al. 2022) hypothesized that a thalidomide analog in which the phthalimide moiety can be replaced with benzotriazole. Using a thermophoresis‐based assay, the authors demonstrated that the “benzotriazolo thalidomide” (4) (Figure 2) has a similar binding mode to thalidomide and the compound displayed relatively stronger binding affinity than thalidomide (1). As well documented in the literature, benzotriazoles, having low lipophilicity, weak basicity, a propensity for poor iron coordination, and thus devoid of any off‐target activity such as CYP inhibition, are a valuable class of heterocyclic compounds with diverse pharmacological properties and synthetic versatility, making them an important tool in medicinal chemistry for drug discovery and development (Briguglio et al. 2014; Shafi'I et al. 2024; Suma et al. 2011). Considering that and with an aim to identify new potent CRBN ligands, we thus divulge here in the design and synthesis of a few benzotriazolo thalidomide derivatives followed by their systematic biological evaluation and thereby showcasing the potential for their use either in PROTAC chemical space or identification of next generation CELMoDs for targeted protein degradation.

## 2 Results and Discussion

### 2.1 Chemistry

In view of the above, compounds 5–13 (Figure 2) were synthesized (see Supporting Information) and screened in various biological assays to identify new CRBN ligands.

The new compounds 5–12 containing either a piperazine or amino or carboxyl substituted piperidine unit envisaged, which can act as linkers for the synthesis of thalidomide‐based PROTACs, whereas compound 13 was derived based on CC‐220 (Matyskiela et al. 2018), which can have additional interactions with cereblon for tighter binding with the receptor and be utilized as a promising starting point to develop a new generation of CELMoDs.

### 2.2 Biological Studies

#### 2.2.1 CRBN/DDB1 Binding Study

Fluorescence polarisation (FP) based CRBN/DDB1 binding assay was performed for the new compounds 5–13, including compound 4, and thalidomide (1) as internal standard, at single point test concentration of 3 μM first. The results are summarized in Table 1. Thalidomide 1 illustrated (80.5% ± 1.8%) inhibition of Cy5 probe binding to CRBN/DDB1 protein whereas benzotriazolo thalidomide 4 demonstrated comparatively lower inhibition (68.5% ± 1.6%), which can be attributed to the use of different assay platforms.

**TABLE 1:** % Inhibition of Cy5 probe binding to CRBN/DDB1 protein.

Compound	% Inhibition at 3 μM	% Inhibition at 1 μM	IC_50_ (nM)
1	80.5 ± 1.8	64.9 ± 3.5	320.2 ± 69.9
4	68.5 ± 1.6		1177 ± 290
5	58.2 ± 2.2		
6	84.9 ± 1.3	63.2 ± 2.5	
7	67.7 ± 3.6		
8	72.1 ± 2.5		
9	82.1 ± 2.7	62.6 ± 1.0	
10	89.3 ± 1.1	79.3 ± 1.1	384 ± 26
11	86.8 ± 4.9	70.8 ± 1.0	540 ± 14
12	82.4 ± 0.4	73.5 ± 1.0	603 ± 205
13	91.4 ± 0.4	74.8 ± 1.4	198 ± 7

*Note:* % inhibition of probe binding and IC_50_ values are the means of independent experiments (*n* = 3).

Towards our objective of identifying more potent CRBN ligands having a benzotriazolo thalidomide core, we wanted to check the effect of a few polar hydrophilic groups at different positions. Thus, the insertion of piperazine and piperidine‐4‐carboxylic acid at the 5‐position of 4 as in compounds 5 and 7 did not improve the inhibition of the probe binding to the protein compared to 4; however, the introduction of the 4‐amino piperidine unit at the same position (compound 6) resulted in better inhibition of the probe binding to the protein than 4 and was similar to thalidomide (1). To our delight, substitution at the 4‐position of the benzotriazolo thalidomide core showcased a beneficial effect as observed for compounds 8 and 9 (72%–82% inhibition of the probe binding to the protein respectively). Interestingly, when the monocyclic unit in compound 8 was replaced by a rigid spiro bicyclic system as in compound 10, higher inhibition of the probe binding towards the CRBN/DDB1 protein was observed under the standard assay conditions, and further substitutions as in compounds 11 and 12 were found to be equally tolerable. On the other hand, compound 13 having a longer hydrophobic side chain linker offered the best impact with > 90% inhibition of Cy5 binding to the CRBN/DDB1 protein. Next, considering significant inhibition of Cy5 binding observed for compounds 6, 9–13, and thalidomide at 3 μM, we wanted to check the dose response effect at 1 μM for these compounds.

Pleasingly, compounds 10–13 demonstrated > 70% inhibition of Cy5 binding to E3 ligase even at 1 μM whereas compounds 1, 6 and 9 offered comparatively lower inhibition of Cy5 binding.

**FIGURE 3:**
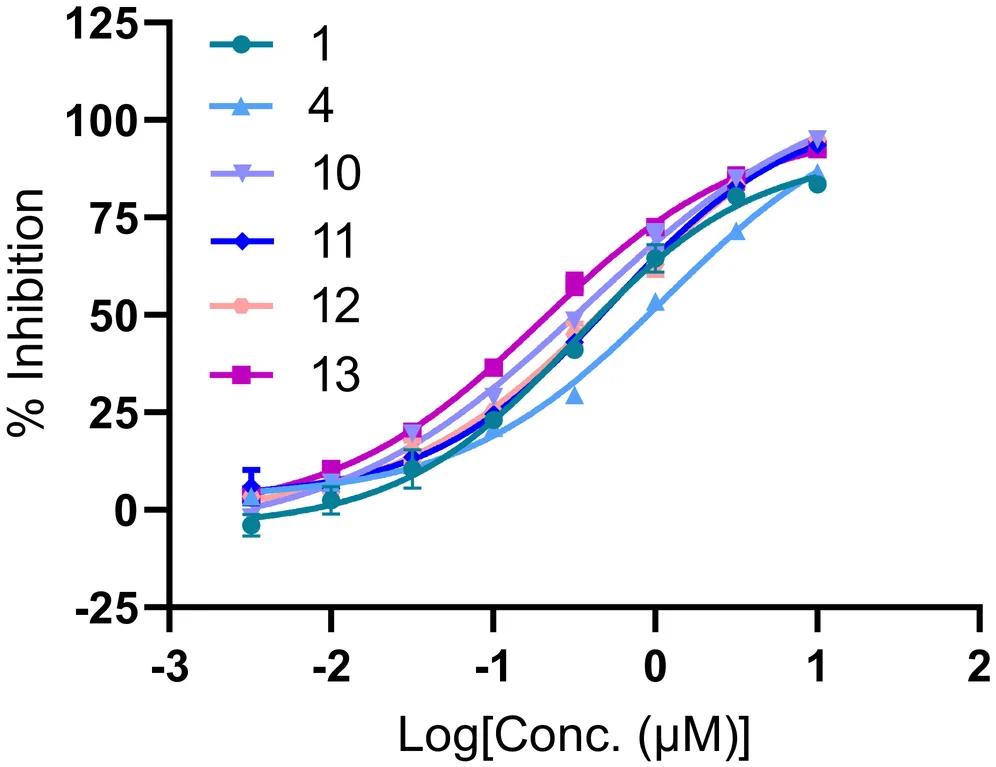
Compiled dose response graphs of test compounds 1, 4, 10–13.

Accordingly, dose response experiment was conducted for compounds 10–13 (along with 1 and 4 for comparison). The data revealed that compound 13 is the most potent CRBN ligand having improved potency than compounds 1 and 4, whereas the benzotriazolothalidomide derivatives 10–12 had IC_50_ values of 384 nM, 540 nM, and 603 nM, respectively. Representative graphs of the test compounds 1, 4, and 10–13 are illustrated in Figure 3.

**FIGURE 4:**
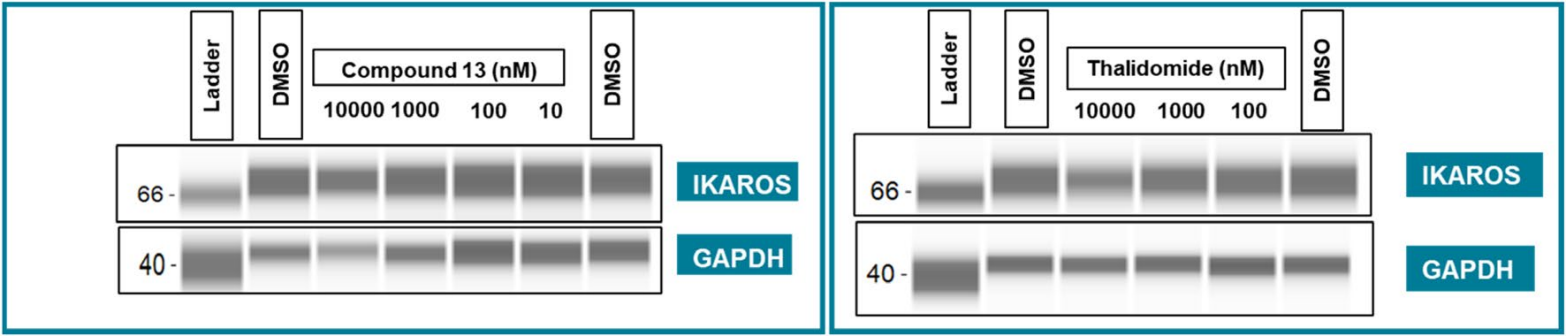
Ikaros degradation study by thalidomide 1 and 13.

Based on the biochemical results of 13, we wanted to study next the effect of the compound in cellular (SU‐DHL‐10 cell) degradation assays measuring the ligand‐dependent depletion of Ikaros. We used a cell‐based assay where the total protein content for each sample was measured using the BCA method (see Supporting Information) and equal amounts of protein were loaded for analysis of the target protein. Ikaros protein in the cell lysate was detected by the Simple Western method using anti‐Ikaros antibody. The total amount of target protein was normalized by the GAPDH protein in respective samples, and treated samples were compared with that of vehicle samples. We find that treatment with compound 13 results in no significant loss of Ikaros protein levels (Figure 4a, see also Supporting Information). Thalidomide was used as reference standard and also demonstrated no degradation (Figure 4b, see also Supporting Information), which is in line with literature precedence (Krönke et al. 2014).

#### 2.2.2 Cell Viability, Metabolic Stability, Solubility, and Lipophilicity of Compound 13

**FIGURE 5:**
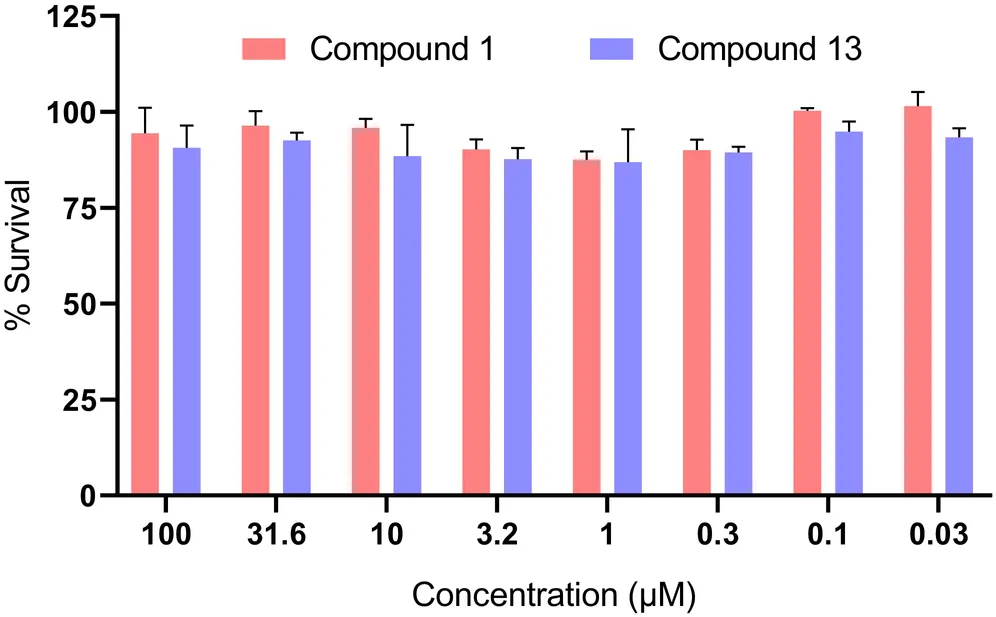
The anti‐proliferative activity of compounds 1 and 13.

Encouraged by the results above of compound 13 we chose to carry out extensive studies for this molecule. Accordingly, cellular viability was measured using CellTiter‐Glo reagent in human embryonic kidney cells (HEK293), treated with the compound in vitro for 72 h, and meager inhibition of cell growth was observed even at the highest dose tested (Figure 5).

The drug‐likeness of compound 13 was characterized next by testing human liver microsomes (hLM) stability, kinetic solubility, and lipophilicity (Kumar et al. 2025). The data (Table 2) revealed that compound 13 was metabolically stable like thalidomide, has a LogD value of 1.81 + 0.01, and excellent aqueous solubility (161.87 + 0.96 μM).

**TABLE 2:** ADME profile of compounds 1 and 13.

Compound	Human liver microsomal stability	Aq. solubility (μg/ml, at pH 7.4)	Lipophilicity (LogD)
	CL_int_, app (μl/min/mg of protein)^a^; T_1/2_ (min)^b^		
13	16.57 ± 0.19; 104.57 ± 1.22	161.87 ± 0.96	1.81 ± 0.01
1	< 14.44; > 120.0	—	—

^a^ Intrinsic clearance.

^b^ Half‐life; the results are mean of independent experiments (*n* = ≥ 2).

### 2.3 Docking Experiments

**FIGURE 6:**
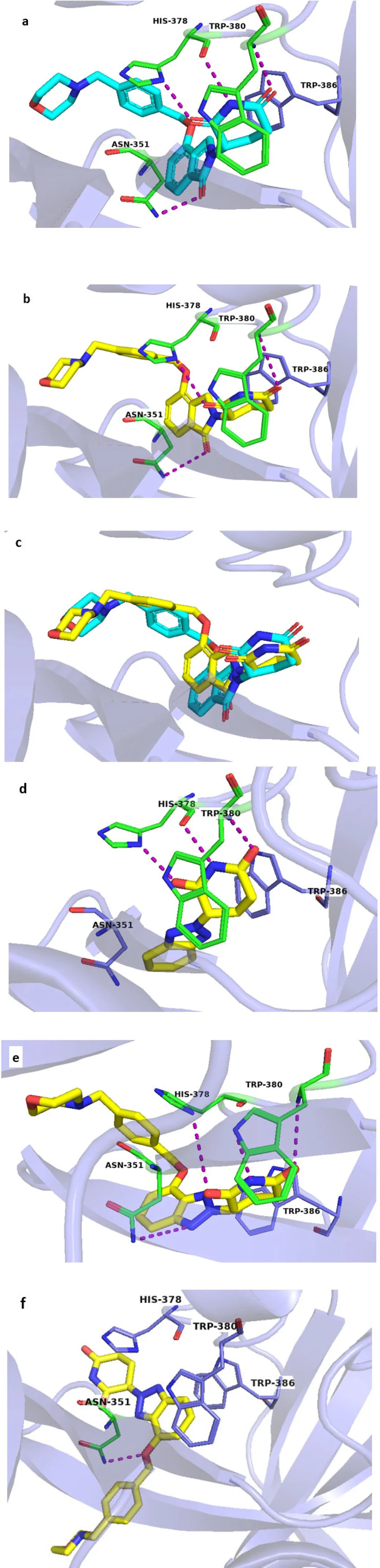
(a) Experimental binding mode of CC‐220; (b) Docked pose of CC‐220 with interactions; (c) Overlapped poses of CC‐220 showing comparison between crystal‐bound (cyan) and docked (yellow) poses; (d) Docked pose of compound 4; (e) Docked pose of compound 13 *S*; (f) Docked pose of compound 13 *R* (all docked poses are in yellow stick model, crystal‐bound ligand in cyan stick model, interacting residues are highlighted in green line model, other non‐interacting neighboring residues in slate line model, overall protein is in slate colored transparent cartoon model and H‐bonds are shown in purple dotted lines).

Towards our future objective of SAR around compound 13 to identify an optimized compound, the putative binding mode of 13 was examined such that the information can be utilized for structure‐based drug design of new CELMoDs. We thus chose the co‐crystal structure of the human cereblon in complex with DDB1 (DNA damage binding protein 1) (5V3O) (Matyskiela et al. 2018) and used it for our docking studies. Its structural analysis revealed that the crystal‐bound ligand CC‐220 is embedded in the tri‐tryptophan pocket and the morpholine group is exposed outside the binding cavity. This compound makes H‐bonding interactions with Asn351 with its C=O group on the isoindolinone and with His378 and Trp380 through the —NH and C=O groups on the glutarimide moiety (Figure 6a). We validated the self‐docking of CC‐220 using our in‐house algorithm (Kumar et al. 2025) taking the chain C from 5V3O, which represents the cereblon part and the bound ligand in it, and both the protein and CC‐220 were hydrogen assigned, and energy minimized in Accelrys Discovery Studio Visualizer. Overall, the docked molecule made H‐bonding interactions with His378 and Trp380 through its two C=O groups on the glutarimide moiety and with Asn351 with the C=O group on the 6–5 system, that is, isoindolinone part (Figure 6b), and the self‐docked pose was found to be similar to the bound ligand pose (Figure 6c) having a binding energy of ˗32.17 kcal/mol. Then, compound 4 was docked to identify its binding mode, which docked with a binding energy of (˗28.19 kcal/mol) and had H‐bond interactions with His378 and Trp380 through its C=O groups and NH part of the glutarimide moiety (Figure 6d). Next, putative binding modes of *S‐* and *R*‐isomers of compound 13 (our docking algorithm can consider stereospecific isomers only) were examined. The docking results of the *S*‐isomer suggest that the compound makes H‐bond interactions with His378 and Trp380 through its C=O groups and NH part of the glutarimide moiety and with Asn351 through the N atom of the benzotriazole moiety (Figure 6e) with a binding energy of ˗31.63 kcal/mol. For compound 13‐*R* (Figure 6f) reduced binding energy of ˗10.2 kcal/mol was observed, indicating no significant binding of the *R* enantiomer in the target site. Based on the findings, we envisage the synthesis of analogs of 13 for detailed structure–activity/structure–property relationships, as our future scope of work.

## 3 Conclusions

In summary, small molecule protein degraders correspond to a new and interesting modality with wide therapeutic potential. Compounds binding to cereblon (CRBN), an E3 ligase, can potentially induce protein–protein interactions with target protein of interest resulting degradation. In this report, we have narrated design, synthesis, in vitro, and docking studies leading to new benzotriazolo thalidomide‐based small molecules, which can interact with the CRL4^CRBN^ E3 ubiquitin ligase substrate receptor CRBN. As a result, focused synthesis and detail biological investigations of a few analogs of compound 4 led to a few potent CRBN ligands among which, compound 13 was found to have high safety level, metabolic stability, and aqueous solubility. These results indicate that the new compounds can be used not only as novel linkers for the synthesis of PROTACs but also can act as promising Hit compound for further optimization towards finding potential in vivo efficacious CELMoDs for the treatment of multiple subtypes of non‐Hodgkin's lymphoma. Structure‐based design of new molecules along with extensive structure activity and structure property relationship studies around compound 13 is our ongoing research interest towards discovering an in vivo efficacious cereblon E3 ligase modulator.

## Author Contributions

**Pramit Ganguly:** investigation, writing – original draft. **Mrinalkanti Kundu:** supervision, conceptualization, writing – original draft. **Tonmoy Sarkar:** investigation. **Gillian Dcosta:** investigation. **Kankana Banerjee:** investigation. **Ritama Banerjee:** investigation. **Arun K. Hajra:** investigation. **Sk Sahabuddin:** investigation. **Sohini Basu:** investigation. **Avratanu Das:** investigation. **Suparna Das:** investigation. **Rajib Barik:** investigation. **Md Ashif Ali:** supervision.

## Funding

The authors have nothing to report.

## Conflicts of Interest

The authors declare no conflicts of interest.

## Supporting information


**Data S1:** cbdd70394‐sup‐0001‐DataS1.docx.

## Data Availability

The data that support the findings of this study are available on request from the corresponding author. The data are not publicly available due to privacy or ethical restrictions.
