# Supplementary material for: Design, Synthesis, Biological Studies and Putative Binding Mode of Novel Benzotriazolothalidomide Derivatives as Cereblon Ligands
Source: Chem Biol Drug Des. 2026 Sep 14;108(3):e70394. doi: 10.1111/cbdd.70394 (PMC13575462; doi:10.1111/cbdd.70394)
Supplement: Supplementary file 1 [file CBDD-108-e70394-s001.docx]

***Supporting information***

**Design, Synthesis, Biological Studies and Putative Binding Mode of Novel Benzotriazolothalidomide Derivatives as Cereblon Ligands**

Pramit Ganguly^1, 2^ | Mrinalkanti Kundu^1*^ | Tonmoy Sarkar^1^ | Gillian Dcosta^1^ | Kankana Banerjee^1^ | Ritama Banerjee ^1^ | Arun K. Hajra ^1^ | Sk Sahabuddin ^1^ | Sohini Basu^1^ | Avratanu Das ^1^ | Suparna Das^1^ | Rajib Barik^1^ | Md Ashif Ali^2*^

^1^TCG Lifesciences Pvt. Ltd., BN- 7, Sector V, Salt Lake, Kolkata- 700091, India.

^2^Department of Chemistry, Ramakrishna Mission Residential College (Autonomous), Narendrapur, Kolkata-700103.

*Corresponding authors

Mrinalkanti Kundu

TCG Lifesciences Pvt. Ltd., BN- 7, Sector V, Salt Lake, Kolkata- 700091, India. E-mail: [mrinal.kundu@tcgls.com](mailto:mrinal.kundu@tcgls.com)

Md Ashif Ali

Department of Chemistry, Ramakrishna Mission Residential College (Autonomous), Narendrapur, Kolkata-700103. E-mail: [ashif.chem@gmail.com](mailto:ashif.chem@gmail.com)

**^1^H NMR Spectroscopy:**

^1^H NMR was recorded at 400.20 & 400.10, 400.17 MHz, on a Bruker Advance, II & III spectrometer using solvents from Merck Laboratories. Chemical shifts (δ, ppm) are reported relative to the solvent peak (CDCl_3_: 7.25, DMSO-*d*6: 2.50). Proton resonances are annotated as: chemical shift (δ), multiplicity (s, singlet; d, doublet; t, triplet; q, quartet; m, multiplet; br, broad), coupling constant (*J*, Hz), and number of protons.

**LCMS methods:**

**Method A (UV 260 and 220 nm):** Column: Waters Acquity UPLC BEH C8 (2.1 x 50 mm, 1.7 u). Mobile phase: 90% [0.05% HCOOH in water] and 10% [0.05% HCOOH in ACN: Water (90:10)] held for 0.75 min, then to 50% [0.05% HCOOH in water] and 50% [0.05% HCOOH in ACN: Water (90:10)  ] in 1.0 min, further to 2% [0.05% HCOOH in water] and 98% [0.05% HCOOH in ACN: Water (90:10)] in 2.0 min, held this mobile phase composition up to 2.25 min and finally back to initial condition in 2.60 min and held this composition up to 3.00 min). Flow =0.80 ml/min.

**Method B (UV 260 and 220 nm):** Column: YMC TRIART C18 (33 x 2.1 mm, 3u). Mobile phase: 98% [0.05% HCOOH in water] and 2% [ACN] held for 0.75 min, then to 90% [0.05% HCOOH in water] and 10% [ACN] in 1.0 min, further to 2% [0.05% HCOOH in water] and 98% [ACN] in 2.0 min, held this mobile phase composition up to 2.25 min and finally back to initial condition in 3.0 min). Flow =1.5 ml/min.

**Method C (UV 260 and 220 nm):** Column: Waters Acquity UPLC BEH C8 (2.1 x 50 mm, 1.7 micron). Mobile phase: 95% [0.05% HCOOH in water] and 5% [0.05% HCOOH in ACN: Water (90:10)] held for 0.75 min, then to 75% [0.05% HCOOH in water] and 25% [0.05% HCOOH in ACN: Water (90:10)  ] in 1.5 min, further to 5% [0.05% HCOOH in water] and 95% [0.05% HCOOH in ACN: Water (90:10)] in 3.00 min, held this mobile phase composition up to 4.00 min and finally back to initial condition in 4.50 min and held this composition up to 5.10 min).

**Method D (DAD Wavelength range (nm): 200 to 400):** Column Used: Waters Acquity UPLC BEH C8 (2.1 x 50 mm, 1.7 micron). (mobile phase: 95% [0.05% HCOOH in water] and 5% [0.05% HCOOH in ACN: Water (90:10)] held for 1.00 min, then 50% [0.05% HCOOH in water] and 50% [0.05% HCOOH in ACN: Water (90:10) ] in 5.00 min, further to 10% [0.05% HCOOH in water] and 90% [0.05% HCOOH in ACN: Water (90:10) ] in 8.00 min, held this mobile phase composition up to 10.00 min and finally back to initial condition in 11.50 min and held this composition up to 12.00 min).

**Method E (UV 260 and 220 nm):** Column Used: Xbridge C18 column (3.5 µm, 50 x 3 mm). (mobile phase: 95% [5 mM NH4OAc in water] and 5% [5 mM NH4OAc in ACN: Water (90:10)] held for 0.75 min, then to 70% [5 mM NH4OAc in water] and 30% [5 mM NH4OAc in ACN: Water (90:10) ] in 1.00 min, and finally 2% [5 mM NH4OAc in water] and 98% [5 mM NH4OAc in ACN: Water (90:10) ] in 2.00 min, held this mobile phase composition up to 2.50 min and finally back to initial condition in 2.75 min and held this composition up to 3.0 min).

**Method E (DAD Wavelength range (nm): 200 to 400):** Column Used: Xbridge C18 column (3.5 µm, 50 x 3 mm): (mobile phase: 95% [5 mM NH4OAc in water] and 5% [5 mM NH4OAc in ACN: Water (90:10)] held for 0.75 min, then 85% [5 mM NH4OAc in water] and 15% [5 mM NH4OAc in ACN: Water (90:10) ] in 1.25 min further 70% [5 mM NH4OAc in water] and 30% [5 mM NH4OAc in ACN: Water (90:10) ] in 2.50 min, and finally 2% [5 mM NH4OAc in water] and 98% [5 mM NH4OAc in ACN: Water (90:10) ] in 3.75 min, held this mobile phase composition up to 4.25 min and finally back to initial condition in 4.50 min and held this composition up to 5.10 min).

**Synthesis of 3-(2H-benzo[d][1,2,3] triazol-2-yl) piperidine-2,6-dione (4):**

**Step-1: Synthesis of 3-(2H-benzo[d][1,2,3] triazol-2-yl) piperidine-2,6-dione (4):** To a stirred solution of 2H-1,2,3-benzotriazole (**I,** 300 mg, 2.52 mmol) in THF (10 mL) was added NaH (252 mg, 6.3 mmol) at 0 °C and the reaction mixture was stirred for 30 min before adding 3-bromopiperidine-2,6-dione (**II**, 532 mg, 2.77 mmol). The reaction mixture was further stirred at rt for 16 h and then quenched with ice water. The aqueous part was then extracted using 10% MeOH/DCM, dried over anhydrous sodium sulphate, evaporated and the crude was purified by reverse prep purification. Two isomers were isolated, the major compound is the desired product (**4,** 150 mg, 26%), for which the structure was confirmed by nOe. ^1^H NMR (400 MHz, MeOD) δ 7.87 (dd, 6.6, 3.1 Hz, 2H), 7.45 (dd, 6.5, 3.0 Hz, 2H), 6.02 – 5.99 (m, 1H), 2.95 – 2.87 (m, 3H), 2.58 – 2.56 (m, 1H). ^13^C NMR (100 MHz, DMSO): δ 172.3, 168.9, 143.6 (2C), 126.8 (2C), 117.9 (2C), 64.7, 30.3, 24.3. LCMS (Method A): RT= 2.31 min; [M+H] ^+^ 231.2. HPLC (UV, λ_210_ _nm_): 99.78%. HRMS (ESI+) calc’d for C11H10N4O2 [M+H] ^+^: 231.0882, found 231.0891.

**Synthesis of 3-(5-(piperazin-1-yl)-2H-benzo[d][1,2,3] triazol-2-yl) piperidine-2,6-dione (5):**

**Step-1: Synthesis of *tert*-butyl 4-(3-amino-4-nitrocyclohexa-1,5-dien-1-yl) piperazine-1-carboxylate (II)**

To a suspension of 5-fluoro-2-nitroaniline (**I**, 3.0 g, 19.22 mmol), *tert*-butyl piperazine-1-carboxylate (3.85 g, 19.22 mmol) and K_2_CO_3_ (2.92 g, 21.14 mmol) in DMF (25 mL) was heated with stirring at 100 °C for 16 h. After completion, the reaction mixture was cooled and diluted with ethyl acetate (50 mL) and washed with water. The aqueous layer was back extracted with ethyl acetate (50 mL) and the combined organic layer was washed with water (100 mL), brine, dried over anhydrous Na_2_SO_4_ and filtered. The filtrate was concentrated under reduced pressure, and the crude material was purified over silica gel column using EtOAc-Hexane (4:1) as eluent to afford *tert-*butyl 4-(3-amino-4-nitrocyclohexa-1,5-dien-1-yl) piperazine-1-carboxylate (**II**, 4.92 g, 80%) as an off white solid. LCMS (Method A): RT= 2.07 min, [M+H]^+^ 323.3.

**Step-2: Synthesis of *tert*-butyl 4-(3,4-diaminophenyl) piperazine-1-carboxylate (III):**

To a stirred solution of   *tert*-butyl 4-(3-amino-4-nitrophenyl) piperazine-1-carboxylate (**II,** 2.0 g, 6.2 mmol) in MeOH (15 mL) was added Zn dust (2.02 g, 31.12 mmol) followed by addition of HCOONH_4_ (1.96 g, 31.12 mmol) at ice cold condition. After 2h, the reaction mixture was filtered through a pad of celite and the filtrate evaporated to dryness to get crude *tert*-butyl 4-(3,4-diaminophenyl) piperazine-1-carboxylate **(III,** 1.25 g, 69%) which was used in the next step without further purification.

^1^H NMR (400 MHz, DMSO) δ 6.39 (d, *J* = 8.2 Hz, 1H), 6.22 (d, *J* = 2.2 Hz, 1H), 6.03 (dd, *J* = 2.2, 8.2 Hz, 1H), 4.37 (s, 2H), 4.02 (s, 2H), 3.40 (s, 4H), 2.81 – 2.79 (m, 4H), 1.40 (s, 9H).LCMS (Method A): RT= 1.59 min, [M+H] ^+^ 293.2

**Step-3: Synthesis of *tert*-butyl 4-(2H-1,2,3-benzotriazol-5-yl) piperazine-1-carboxylate (IV):**

*tert*-butyl 4-(3,4-diaminophenyl) piperazine-1-carboxylate (**III,** 1.24 g, 4.26 mmol) was added to a flask containing ice cold glacial acetic acid (30.0 mL). A solution of NaNO_2_ (323.7 mg, 4.69 mmol) in water (30 mL) was added to the reaction mixture drop wise and stirred at room temperature for 30 min. The reaction mixture filtered, and the filtrate was concentrated under reduced pressure. The crude material was dissolved in ethyl acetate (25 mL) and washed with water (25 mL). The aqueous layer was back extracted with ethyl acetate (25 mL) and the combined organic layer was washed with water (25 mL), brine, dried over anhydrous Na_2_SO_4_ and filtered. The filtrate was concentrated under reduced pressure to get crude residue, which was purified by silica gel using EtOAc-hexane (1:4) as eluent to get *tert*-butyl 4-(2H-1,2,3-benzotriazol-5-yl) piperazine-1-carboxylate (**IV,** 770 mg, 60%) as an off white solid.

^1^H NMR (400 MHz, DMSO) δ 15.28 (s, 1H), 7.79 (d, *J* = 9.1 Hz, 1H), 7.22 (d, *J* = 9.1 Hz, 1H), 7.03 (s, 1H), 3.49 (s, 4H), 3.17 (4, 4H), 1.42 (s, 9H). LCMS (Method A): RT= 1.87 min; [M+H] ^+^ 304.2

**Step-4: Synthesis of *tert*-butyl 4-[2-(2,6-dioxopiperidin-3-yl)-2H-1,2,3-benzotriazol-5-yl] piperazine-1-carboxylate (VI)**

To an ice-cold solution of NaH (60 wt% in mineral oil, 276 mg, 6.9 mmol) in THF (5.0 mL) was added *tert*-butyl 4-(2H-1,2,3-benzotriazol-5-yl) piperazine-1-carboxylate (**IV,** 700.0 mg, 2.3 mmol) at 0°C and stirred at room temperature for 20 min. A solution of 3-bromopiperidine-2,6-dione (**V,** 886.1 mg, 4.6 mmol) in THF (5 mL) was added and the resulting reaction mixture was stirred at room for 4 h. The reaction mixture was poured into sat aqueous NH_4_Cl. The reaction mixture was diluted with ethyl acetate (10 mL) and washed with water (20 mL). The aqueous layer was back extracted with ethyl acetate (10 mL) and the combined organic layer was washed with water (20 mL), brine, dried over anhydrous Na_2_SO_4_ and filtered. The filtrate was concentrated under reduced pressure, and the crude material was purified over silica gel using EtOAc-Hexane (1:3) as eluent to afford *tert*-butyl 4-[2-(2,6-dioxopiperidin-3-yl)-2H-1,2,3-benzotriazol-5-yl]piperazine-1-carboxylate (**VI,** 420 mg, 60%) as an off white solid.

^1^H NMR (400 MHz, DMSO) δ 11.23 (s, 1H), 7.78 (d, *J* = 9.3 Hz, 1H), 7.38 (dd, *J* = 1.7, 9.4 Hz, 1H), 7.08 (d, *J* = 1.2 Hz, 1H), 6.04 - 5.99 (m, 1H), 3.49 (s, 4H), 3.16 - 3.14 (m, 4H), 2.89 – 2.67 (m, 3H), 2.45 - 2.41 (m, 1H), 1.43 (s, 9H). LCMS (Method A): RT= 1.93 min; [M+H] ^+^ 415.2

**Step-5: Synthesis of 3-(5-(piperazin-1-yl)-2H-benzo[d][1,2,3] triazol-2-yl) piperidine-2,6-dione (5):**

A solution of   *tert*-butyl 4-[2-(2,6-dioxopiperidin-3-yl)-2H-1,2,3-benzotriazol-5-yl] piperazine -1-carboxylate (**VI,** 35 mg, 0.08 mmol) in DCM (2 mL) was treated with HCl in Ether (2M) (0.064 mL, 0.84 mmol) for 2h. The solvent was removed under vacuo, and the crude material was triturated with diethyl ether and pentane to get 3-(5-(piperazin-1-yl)-2H-benzo[d] [1,2,3] triazol-2-yl) piperidine-2,6-dione as hydrochloride salt (**5,** 26 mg, 90%) as an off white solid.

^1^H NMR (400 MHz, DMSO): δ 11.24 (s, 1H), 9.09 (br s, 2H), 7.83 (d, *J* = 9.4 Hz, 1H), 7.38 (dd, *J* = 1.7, 9.4 Hz, 1H), 7.18 (s, 1H), 6.06 - 6.02 (m, 1H), 3.43 - 3.42 (m, 4H), 3.24-3.28 (m, 4H), 2.93 – 2.67 (m, 3H), 2.44 - 2.42 (m, 1H). ^13^C NMR (100 MHz, DMSO): δ 172.4, 169.0. 149.3, 144.8, 139.7, 122.2, 118.3, 99.5, 64.3, 46.2, 42.4, 30.3, 24.4. LCMS (Method A): RT= 0.41 min; [M+H] ^+^ 315.2. HPLC (UV, λ_210_ _nm_): 98.25%. HRMS (ESI+) calc’d for C15H18N6O2 [M+H] ^+^: 315.1569, found 315.1582

**Synthesis of 3-(5-(4-aminopiperidin-1-yl)-2H-benzo[d][1,2,3]triazol-2-yl)piperidine-2,6-dione hydrochloride (6):**

**Step-1:  Synthesis of *tert*-butyl (1-(3-amino-4-nitrophenyl) piperidin-4-yl) carbamate (III):**

A mixture of 5-fluoro-2-nitroaniline (**I,** 3.0g, 19.21 mmol), tert-butyl N-(piperidin-4-yl)carbamate (**II,** 3.85 g, 19.21 mmol) and [K_2_CO_3_](https://www.commonorganicchemistry.com/Common_Reagents/Potassium_Carbonate/Potassium_Carbonate.htm) (7.97 g, 57.65 mmol) in D[M](https://www.commonorganicchemistry.com/Common_Reagents/N-Methyl-2-pyrrolidone/N-Methyl-2-pyrrolidone.htm)F (30 mL) was stirred at 80 ℃ for 16 h. After completion, the reaction mixture was cooled and diluted with ethyl acetate (30 mL) and washed with water (30 mL). The aqueous layer was back extracted with ethyl acetate (30 mL) and the combined organic layer was washed with water (60 mL), brine, dried over anhydrous Na_2_SO_4_, filtered. The filtrate was concentrated under reduced pressure, and the crude material was purified by combi flash using EtOAc-hexane (1:4) as eluent to afford *tert*-butyl (1-(3-amino-4-nitrophenyl) piperidin-4-yl) carbamate (**III,** 6.0 g, 94%) as yellow solid.

^1^H NMR (400 MHz, DMSO) δ 7.79 (d, *J* = 9.7 Hz, 1H), 7.22 (s, 2H), 6.87 (d, *J* = 7.4 Hz, 1H), 6.36 (d, *J* = 9.7 Hz, 1H), 6.20 (s, 1H), 3.80 (d, *J* = 13.2 Hz, 2H), 3.54 – 3.49 (m, 1H), 2.97 (t, *J* = 11.9 Hz, 2H), 1.82 – 1.74 (m, 2H), 1.47-1.23 (m, 11H). LCMS (Method A): RT= 2.04 min; [M+H] ^+^ 337.3

**Step-2:  Synthesis of *tert*-butyl (1-(3-amino-4-nitrophenyl) piperidin-4-yl) carbamate (IV):**

To an ice-cold stirred solution of    *tert*-butyl N-[1-(3-amino-4-nitrophenyl) piperidin-4-yl] carbamate (**III,** 6.0 g, 8.92 mmol) in MeOH (25 mL) was added Zn dust (2.9 g, 44.59 mmol) followed by the addition of HCOONH_4_ (2.81 g, 44.59 mmol). After 2h of stirring, the reaction mixture was filtered through a pad of celite and the filtrate was evaporated to get N-[1-(3,4-diaminophenyl) piperidin-4-yl] carbamate (**IV,** 3.0 g, 56%) as an off white solid, which was used in the next step without further purification.

^1^H NMR (400 MHz, DMSO) δ 6.81 (d, *J* = 7.5 Hz, 1H), 6.37 (d, *J* = 8.2 Hz, 1H), 6.2 (s, 1H), 6.00 (d, *J* = 8.4 Hz, 1H), 4.33 (s, 2H), 3.96 (s, 2H), 3.28-3.25 (m, 3H), 2.47 – 2.40 (m, 2H), 1.76 – 1.73 (m, 2H), 1.54 – 1.43 (m, 2H), 1.38 (s, 9H). LCMS (Method A): RT= 1.59 min; [M+H] ^+^ 307.3.

**Step-3:** **Synthesis of *tert*-butyl (1-(2H-benzo[d][1,2,3] triazol-5-yl) piperidin-4-yl) carbamate (V):**

To an ice-cold stirred solution of   *tert*-butyl N-[1-(3,4-diaminophenyl) piperidin-4-yl] carbamate (**IV,** 3.0 g, 9.79 mmol) in AcOH (30. mL) was added an aqueous solution (30 mL) of NaNO_2_ (0.74 g, 10.77 mmol) drop wise. Reaction mass was stirred for 30 mins, after completion the reaction mixture was concentrated, and the reaction mixture was diluted with ethyl acetate and washed with water. The aqueous layer was back extracted with ethyl acetate, and the combined organic layer was washed with water, brine, dried over anhydrous sodium sulfate, filtered. The filtrate was concentrated under reduced pressure, and the crude material was purified over silica gel using EtOAc-hexane (1:5) as eluent to afford *tert*-butyl (1-(2H-benzo[d][1,2,3] triazol-5-yl) piperidin-4-yl) carbamate (**V,** 1.55 g, 50%) as off white solid.

^1^H NMR (400 MHz, DMSO) δ 15.14 (brs, 1H), 7.78 (br s, 1H), 7.16 (br s, 1H), 6.86 (d, J = 7.3 Hz, 1H), 3.69 (br s, 2H), 3.41 (br s, 1H), 2.79 – 2.77 (m, 2H), 1.83 – 1.81 (m, 2H), 1.54 – 1.49 (m, 2H), 1.39 (s, 9H). LCMS (Method A): RT= 1.75 min; [M+H] ^+^ 318.3.

**Step-4: Synthesis of *tert*-butyl (1-(2-(2,6-dioxopiperidin-3-yl)-2H-benzo[d][1,2,3] triazol-5-yl) piperidin-4-yl) carbamate (VII):**

Under argon atmosphere, NaH (53 mg, 1.32 mmol) was added to an ice-cold stirred solution *tert*-butyl 4-(2H-1,2,3-benzotriazol-5-yl) piperazine-1-carboxylate (**V,** 200.0 mg, 0.66 mmol) in THF (5.0 mL)    and the resulting reaction mixture was stirred at rt for 30 mins. 3-Bromopiperidine-2,6-dione (**VI,** 254 mg, 1.32 mmol) was added and the reaction mixture was allowed to come at room temperature. After completion, the reaction mixture was quenched with careful addition of saturated solution of NH_4_Cl and the reaction mixture was diluted with ethyl acetate (10 mL) and washed with water. The aqueous layer was back extracted with ethyl acetate (10 mL) and the combined organic layer was washed with water, brine, dried over anhydrous sodium sulfate, filtered. The filtrate was concentrated under reduced pressure, and the crude material was purified by Prep-TLC using 30% EtOAc-hexane as mobile phase to afford **(VII,** 150 mg 55%)

^1^H NMR (400 MHz, DMSO) δ 11.22 (s, 1H), 7.78 (d, *J* = 9.4 Hz, 1H), 7.38 (dd, *J* = 1.7, 9.4 Hz, 1H), 7.09 (s, 1H), 6.01 (dd, *J* = 5.0, 11.8 Hz, 1H), 3.50 (s, 4H), 3.39 – 3.34 (m, 2H), 3.28 – 3.26 (m, 1H), 3.15 (s, 4H), 2.89 - 2.68 (s, 3H), 2.45 - 2.41 (m, 1H), 1.42 (s, 9H). LCMS (Method A): RT= 1.53 min; [M+H] ^+^ 429.3.

**Step-5:** **Synthesis of 3-(5-(4-aminopiperidin-1-yl)-2H-benzo[d][1,2,3] triazol-2-yl) piperidine-2,6-dione (6):**

To an ice-cold solution of *tert*-butyl N-{1-[2-(2,6-dioxopiperidin-3-yl)-2H-1,2,3-benzotriazol-5-yl] piperidin-4-yl} carbamate (**VII**, 250 mg, 0.583 mmol) in DCM (5 mL) was added 4M HCl in dioxane (5 mL). A grey precipitate formed after 4 h. The solvent was removed under vacuo, and the resultant grey solid was triturated with diethyl ether and pentane to afford 3-(5-(4-aminopiperidin-1-yl)-2H-benzo[d][1,2,3] triazol-2-yl) piperidine-2,6-dione (**6,** 150 mg, 70%).

^1^H NMR (400 MHz, DMSO) δ 11.25 (s, 1H), 8.22 (br s, 3H), 7.83 (d, *J* = 9.4 Hz, 1H), 7.48 (d, *J* = 8.8 Hz, 1H), 7.31 (br s, 1H), 6.05 (dd, J = 5.0, 11.8 Hz, 1H), 3.82 - 3.79 (m, 2H), 3.26 (br s, 1H), 2.95 – 2.68 (m, 5H), 2.46 - 2.42 (m, 1H), 2.06 - 2.03 (s, 2H), 1.79 - 1.76 (m, 2H).^13^C NMR (100 MHz, DMSO): δ 172.4, 168.9, 143.7, 141.4, 121.7, 119.4, 64.9, 50.7, 45.5, 30.3, 27.8, 24.4. LCMS (Method C): RT= 1.63 min; [M+H]^+^ 329.3. HPLC (UV, λ_210_ _nm_): 98.53%. HRMS (ESI+) calc’d for C16H20N6O2 [M+H]^+^: 329.1726, found 329.1724

**Synthesis of 1-(2-(2,6-dioxopiperidin-3-yl)-2H-benzo[d][1,2,3]triazol-5-yl)piperidine-4-carboxylic acid (7):**

**Step-1: *tert*-butyl 1-(3-amino-4-nitrophenyl) piperidine-4-carboxylate (III):**

A solution of 5-fluoro-2-nitroaniline (**I,** 3.1 g, 19.86 mmol) and tert-butyl piperidine-4-carboxylate (**II,** 4.05 g, 21.84 mmol) in DMF (25 mL)   was added K_2_CO_3_ (3.01 g, 21.84 mmol) and the resulting suspension was heated at 80 ^0^C for 16h. The reaction mixture was cooled and filtered; the filtrate was diluted with ethyl acetate and washed with water. The aqueous layer was back extracted with ethyl acetate, and the combined organic layer was washed with water, brine, dried over anhydrous sodium sulphate, filtered and the filtrate was concentrated under reduced pressure to get the crude material which was purified by silica gel column chromatography using 30-50% EtOAc-Hexane as eluent to afford *tert*-butyl 1-(3-amino-4-nitrophenyl) piperidine-4-carboxylate (**III**, 3.5 g 54%)

^1^H NMR (400 MHz, DMSO) δ 7.79 (d, *J* = 9.7 Hz, 1H), 7.24 (s, 2H), 6.37 (d, *J* = 9.8 Hz, 1H), 6.21 (s, 1H), 3.79 – 3.76 (m, 2H), 3.02 - 2.88 (m, 3H), 1.85 – 1.80 (m, 2H), 1.56 - 1.48 (m, 2H), 1.39 (s, 9H). LCMS (Method A): RT= 2.23 min; [M+H] ^+^ 322.3

**Step-2: *tert*-butyl 1-(3,4-diaminophenyl) piperidine-4-carboxylate (IV):**

To a stirred solution of *tert*-butyl 1-(3-amino-4-nitrophenyl) piperidine-4-carboxylate (**III,** 100 mg, 0.31 mmol) in methanol (5 mL)   was added Zn dust (101.13 mg, 1.56 mmol) followed by the addition of HCOONH_4_ (98.02 mg, 1.56 mmol) and the resulting reaction mixture was stirred for 16h. After completion of the reaction mixture was filtered off and the volatiles were removed under reduced pressure to dryness. The crude material (**IV,** 70 mg 77%) was thus obtained was used in the next step without further purification.

^1^H NMR (400 MHz, DMSO) δ 6.38 (d, *J* = 8.2 Hz, 1H), 6.2 (s, 1H), 6.00 (d, *J* = 8.2 Hz, 1H), 4.24 (br s, 4H), 3.27 – 3.24 (m, 2H), 2.53 - 2.48 (m, 2H), 2.27 – 2.21 (m, 1H), 1.83 - 1.80 (m, 2H), 1.65 - 1.56 (m, 2H), 1.40 (s, 9H). LCMS (Method A): RT= 1.61 min; [M+H] ^+^ 292.3

**Step-3: *tert*-butyl 1-(2H-1,2,3-benzotriazol-5-yl) piperidine-4-carboxylate (V):**

To an ice-cold stirred solution of   *tert*-butyl 1-(3,4-diaminophenyl) piperidine-4-carboxylate (**IV,** 1.4 gm, 4.8 mmol) in   glacial acetic acid (30 mL) was added a solution of NaNO_2_ (0.36 gm, 5.29 mmol) in   water (30 mL) drop wise. Reaction mass was stirred at rt for 30 mins, after completion the reaction mixture was concentrated, diluted with EtOAc, washed with aqueous NaHCO_3_ solution, brine, dried over anhydrous Na_2_SO_4_, filtered and the filtrate was concentrated to dryness. The crude material was purified by combi flash using 30-50%EtOAc-hexane as eluent to afford *tert*-butyl 1-(2H-1,2,3-benzotriazol-5-yl) piperidine-4-carboxylate (**V,** 1.1 g, 64%) as an off white solid. ^1^H NMR (400 MHz, DMSO) δ 7.75 (d, *J* = 9.2 Hz, 1H), 7.19 (d, *J* = 9.2 Hz, 1H), 6.98 (s, 1H), 3.68 – 3.65 (m, 2H), 2.84 - 2.78 (m, 2H), 2.46 – 2.37 (m, 2H), 1.91 - 1.85 (m, 2H), 1.69 - 1.64 (m, 2H), 1.41 (s, 9H). LCMS (Method A): RT= 2.02 min; [M+H] ^+^ 303.3

**Step-4: Synthesis of *tert*-butyl 1-[2-(2,6-dioxopiperidin-3-yl)-2H-1,2,3-benzotriazol-5-yl] piperidine-4-carboxylate (VII):**

To an ice cold solution of the *tert*-butyl 1-(2H-1,2,3-benzotriazol-5-yl)piperidine-4-carboxylate (**V,** 1.0 g, 3.3 mmol) in [THF](https://www.commonorganicchemistry.com/Common_Reagents/Tetrahydrofuran/Tetrahydrofuran.htm) (20 mL) was added [NaH](https://www.commonorganicchemistry.com/Common_Reagents/Sodium_Hydride/Sodium%20Hydride.htm) (396 mg, 9.9 mmol) portion wise and the resulting mixture was stirred at room temperature for 15 min. To the reaction mass was then added 3-bromopiperidine-2,6-dione (**VI,** 238 mg, 9.9 mmol) and stirring was continued for 16 h. The mixture was quenched by the addition of saturated aqueous NH_4_Cl, then diluted with [EtOAc](https://www.commonorganicchemistry.com/Common_Reagents/Ethyl_Acetate/Ethyl%20Acetate.htm). The organic layer was separated and the aqueous layer was further extracted with [EtOAc](https://www.commonorganicchemistry.com/Common_Reagents/Ethyl_Acetate/Ethyl%20Acetate.htm). The combined organics were washed with brine, dried over anhydrous Na_2_SO_4_, and concentrated under vacuo*.* The resulting crude material was purified by flash chromatography (eluting with 50-70% [EtOAc](https://www.commonorganicchemistry.com/Common_Reagents/Ethyl_Acetate/Ethyl%20Acetate.htm)/[heptane](https://www.commonorganicchemistry.com/Common_Reagents/Heptane/Heptane.htm)) to provide *tert*-butyl 1-[2-(2,6-dioxopiperidin-3-yl)-2H-1,2,3-benzotriazol-5-yl]piperidine-4-carboxylate (**VII,** 410 mg, 30%) as a yellow solid.

^1^H NMR (400 MHz, DMSO) δ 11.24 (s, 1H), 7.74 (d, *J* = 9.4 Hz, 1H), 7.35 (d, *J* = 9.5 Hz, 1H), 7.03 (s, 1H), 6.05 -5.96 (m, 1H), 3.79 – 3.53 (m, 2H), 2.99 - 2.64 (m, 5H), 2.46 - 2.34 (m, 2H), 1.94 - 1.87 (m, 2H), 1.86 - 1.74 (m, 1H), 1.74 - 1.61 (m, 2H), 1.44 - 1.37 (m, 9H). LCMS (Method A): RT= 1.97 min; [M+H]^+^ 414.3

**Step-5: Synthesis of 1-(2-(2,6-dioxopiperidin-3-yl)-2H-benzo[d][1,2,3] triazol-5-yl) piperi dine-4-carboxylic acid (7):**

To a stirred solution of *tert*-butyl 1-[2-(2,6-dioxopiperidin-3-yl)-2H-1,2,3-benzotriazol-5-yl]piperidine-4-carboxylate (**VII,** 250.0 mg, 0.61 mmol) in DCM (5 mL) was added 4M [HCl](https://www.commonorganicchemistry.com/Common_Reagents/Hydrochloric_Acid/Hydrochloric_Acid.htm) in [dioxane](https://www.commonorganicchemistry.com/Common_Reagents/Dioxane/Dioxane.htm) (5 mL). A precipitate formed after 4 h. Diethyl ether was added, and the resultant solid was filtered and triturated with pentane to afford 1-(2-(2,6-dioxopiperidin-3-yl)-2H-benzo[d][1,2,3] triazol-5-yl) piperidine-4-carboxylic acid (**7,** 130 mg, 60%) as an off white solid.

^1^H NMR (400 MHz, DMSO) δ 11.24 (s, 1H), 7.79 (d, *J* = 9.4 Hz, 1H), 7.42 (dd, *J* = 9.4, 2.0 Hz, 1H), 7.29-7.12 (m, 1H), 6.02 (dd, J = 11.7, 5.1 Hz, 1H), 3.67 (d, J = 12.6 Hz, 2H), 2.88 - 2.67 (m, 5H), 2.50 - 2.41 (m, 2H), 1.98-1.94 (m, 2H), 1.79-1.74 (m, 2H). ^13^C NMR (100 MHz, DMSO): δ 175.8, 172.3, 169.0, 150.5, 145.1, 139.2, 122.7, 117.8, 98.4, 64.1, 48.9, 30.2, 27.6, 24.3. LCMS (Method C): RT= 1.47 min; [M+H]^+^ 358.2. HPLC (UV, λ_210_ _nm_): 99.68%. HRMS (ESI+) calc’d for C17H19N5O4 [M+H]^+^: 358.1515, found 358.1530

**Synthesis of 3-(4-(piperidin-4-yloxy)-2H-benzo[d][1,2,3]triazol-2-yl)piperidine-2,6-dione hydrochloride (Cpd-8) & 3-(4-((1-benzylpiperidin-4-yl)oxy)-2H-benzo[d][1,2,3]triazol-2-yl)piperidine-2,6-dione (9):**

**Step-1: Synthesis of *tert*-butyl 4-(3-amino-2-nitrophenoxy) piperidine-1-carboxylate (III):**

To a well stirred solution of   tert-butyl 4-hydroxypiperidine-1-carboxylate (**I,** 500 mg, 2.48 mmol) in THF (10 mL) was added   NaH (60%) (89.43 mg, 3.73 mmol) at 0°C and stirred at rt for 10 min.  3-fluoro-2-nitroaniline (**II,** 387.83 mg, 2.48 mmol) was added and continued to stir at room temperature for 12h.The reaction was monitored by the LCMS. Reaction mixture was quenched with water and extracted with EtOAc. Organic layer was separated, dried over anhydrous Na_2_SO_4_, filtered, evaporated to get the crude which was purified by combi flash column chromatography to afford *tert*-butyl 4-(3-amino-2-nitrophenoxy) piperidine-1-carboxylate (**III,** 300 mg, 36%).

^1^H NMR (400 MHz, DMSO) δ 7.11 (t, *J* = 8.3 Hz, 1H), 6.43 (d, *J* = 8.3 Hz, 1H), 6.38 (d, *J* = 8.1 Hz, 1H), 5.97 (s, 2H), 4.66 (br s, 1H), 3.43 (brs, 2H), 3.33 - 3.29 (m, 2H), 1.80 (br s, 2H), 1.56 (br s, 2H), 1.40 (s, 9H). LCMS (Method-F): RT= 1.94 min; [M-H]^-^ 336.2

**Step-2: Synthesis of *tert*-butyl 4-(2,3-diaminophenoxy) piperidine-1-carboxylate (IV):**

To a well stirred solution of   *tert*-butyl 4-(3-amino-2-nitrophenoxy) piperidine-1-carboxylate (**III,** 300 mg, 0.89 mmol) in MeOH (8 mL) was added   Zn (290.73 mg, 4.45 mmol), HCOONH_4_ (280.37 mg, 4.45 mmol) and stirred at rt for 2h.  The reaction was monitored by the LCMS. Reaction mixture was filtered on celite bed and washed with EtOAc. The crude was purified by combi flash column chromatography to afford the *tert*-butyl 4-(2,3-diaminophenoxy) piperidine-1-carboxylate (**IV,** 150 mg, 55%).

^1^H NMR (400 MHz, DMSO) δ 6.34 (t, *J* = 7.8 Hz, 1H), 6.23-6.21 (m, 2H), 4.47 (s, 2H), 4.37 - 4.36 (m, 1H), 4.01 (s, 2H), 3.64 - 3.61 (m, 2H), 3.18 - 3.16 (m, 2H), 1.84 - 1.81 (m, 2H), 1.58 - 1.55 (m, 2H), 1.40 (s, 9H). LCMS (Method-E): RT= 1.78 min; [M+H]^+^ 308.2

**Step-3: Synthesis of *tert*-butyl 4-((2H-benzo[d][1,2,3] triazol-4-yl) oxy) piperidine-1-carboxylate (V):**

To a well stirred solution of *tert*-butyl 4-(2,3-diaminophenoxy) piperidine-1-carboxylate (**IV,** 200 mg, 0.65 mmol) in AcOH (2 mL) was added   NaNO_2_ (53.87 mg, 0.78 mmol) in H_2_O (2 mL) at 0°C and stirred at rt for 1h.  The reaction was monitored by the LCMS. Reaction mixture was quenched with water and extracted with EtOAc. Organic layer was washed with saturated NaHCO_3_ and brine solution, dried over anhydrous sodium sulphate, evaporated to get the crude which was purified by combi flash column chromatography to afford (**V**, 100 mg, 48%) the *tert*-butyl 4-(2H-1,2,3-benzotriazol-4-yloxy) piperidine-1-carboxylate.

^1^H NMR (400 MHz, DMSO) δ 15.66 (br s, 1H), 7.30 (br s, 2H), 6.88 (br s, 1H), 5.09 (br s, 1H), 3.70 (br s, 2H), 3.32 - 3.16 (m, 2H), 1.99 (br s, 2H), 1.66 (br s, 2H), 1.42 (s, 9H). LCMS (Method-E): RT= 1.80 min; [M+H]^+^ 319.2

**Step-4: Synthesis of *tert*-butyl 4-((2-(2,6-dioxopiperidin-3-yl)-2H-benzo[d][1,2,3] triazol-4-yl) oxy) piperidine-1-carboxylate (VII):**

To a well stirred solution of *tert*-butyl 4-(2H-1,2,3-benzotriazol-4-yloxy) piperidine-1-carboxylate (**V,** 140 mg, 0.44 mmol) in THF (4 mL) was added   NaH (44 mg, 1.1 mmol) at 0°C and stirred at rt for 10 min.  3-bromopiperidine-2,6-dione (**VI,** 211.08 mg, 1.1 mmol) was added and continued to stir at room temperature for 24h.The reaction was monitored by the LCMS. Reaction mixture was quenched with water and extracted with EtOAc. Organic layer was separated, dried over anhydrous sodium sulphate, evaporated under vacuo to get the crude which was purified by combi flash column chromatography to afford the *tert*-butyl 4-{[2-(2,6-dioxopiperidin-3-yl)-2H-1,2,3-benzotriazol-4-yl] oxy} piperidine-1-carboxylate (**VII**, 90 mg, 48%).

^1^H NMR (400 MHz, DMSO) δ 11.28 (s, 1H), 7.45 (d, *J* = 8.4 Hz, 1H), 7.35 (t, *J* = 8.2, 8.0 Hz, 1H), 6.93 (d, *J* = 7.7 Hz, 1H), 6.14 (d, *J* = 10.7 Hz, 1H), 4.87 (br s, 1H), 3.76 - 3.73 (m, 2H), 3.19 (brs, 2H), 2.93 - 2.67 (m, 3H), 2.50 (1H merged with solvent peak), 2.08 - 1.99 (m, 2H), 1.63 - 1.61 (m, 2H), 1.41 (s, 9H). LCMS (Method-F): RT= 2.63 min; [M-H]^-^ 428.3

**Step-5: Synthesis of 3-(4-(piperidin-4-yloxy)-2H-benzo[d][1,2,3] triazol-2-yl) piperidine-2,6-dione hydrochloride (8):**

To a well stirred solution of *tert*-butyl 4-{[2-(2,6-dioxopiperidin-3-yl)-2H-1,2,3-benzotriazol-4-yl] oxy} piperidine-1-carboxylate (**VII,** 70 mg, 0.16 mmol) in 1,4 Dioxane (1 mL) was added Dioxane-HCl (4M, 1 mL) at 0°C and stirred the mixture at rt for 1h. The reaction was monitored by the LCMS. Diethyl ether was added into reaction mixture and white precipitate was collected by filtration. Solid material was triturated by pentane to afforded 3-[4-(piperidin-4-yloxy)-2H-1,2,3-benzotriazol-2-yl] piperidine-2,6-dione hydrochloride (**8**, 40 mg, 66%).

^1^H NMR (400 MHz, DMSO) δ 11.28 (s, 1H), 8.75 (br s, 2H) 7.49 (d, *J* = 8.5 Hz, 1H), 7.37 (t, J = 7.9 Hz, 1H), 6.97 (d, J = 7.5 Hz, 1H), 6.16 - 6.12 (m, 1H), 4.96 (br s, 1H), 3.32 (br s, 2H merged with solvent peak), 3.12 (br s, 2H), 2.91 - 2.67 (m, 3H), 2.50 (1H merged with solvent peak), 2.20 (brs, 2H), 1.94 - 1.93 (m, 2H). ^13^C NMR (100 MHz, DMSO) δ 172.3, 168.8, 147.0, 145.6, 137.1, 127.5, 110.0, 106.7, 70.0, 64.7, 30.3 (2C), 27.0 (2C), 26.9. LCMS (Method-F): RT= 1.71 min; [M+H]^+^ 330.3. HPLC (UV, λ_220_ _nm_): 93.85%. HRMS (ESI+) calc’d for C16H19N5O3 [M+H]^+^: 330.1566, found 330.1576

**Step-6: Synthesis of  3-{4-[(1-benzylpiperidin-4-yl)oxy]-2H-1,2,3-benzotriazol-2-yl}piperi dine-2,6-dione (9):**

To a well stirred solution of 3-[4-(piperidin-4-yloxy)-2H-1,2,3-benzotriazol-2-yl] piperidine-2,6-dione (**8**, 300 mg, 0.91 mmol) in MeOH (5 mL) was added benzaldehyde (**VIII,** 193.32 mg, 1.82 mmol) and NaCNBH_3_ (114.48 mg, 1.82 mmol) at 0°C and stirred at rt for 16h.  The reaction was monitored by the LCMS. Solvent was removed under reduced pressure and resultant crude was purified by column chromatography to get 3-{4-[(1-benzylpiperidin-4-yl) oxy]-2H-1,2,3-benzotriazol-2-yl} piperidine-2,6-dione (**9,** 80 mg, 23%).

^1^H NMR (400 MHz, DMSO) δ 11.29 (br s, 1H), 7.42 (d, *J* = 8.6 Hz, 1H) 7.34-7.24 (m, 5H), 6.97 (d, *J* = 7.5 Hz, 1H), 6.16 - 6.12 (m, 1H), 4.69 (br s, 1H), 3.51 (s, 2H), 2.89 - 2.67 (m, 5H), 2.50 - 2.49 (m, 1H, merged with solvent peak), 2.33 - 2.24 (m, 2H), 2.04 (br s, 2H), 1.75 - 1.72 (m, 2H). ^13^C NMR (100 MHz, DMSO) δ 172.3, 168.9, 147.7, 145.6, 138.4, 137.3, 128.7, 128.1, 127.6, 126.8, 109.3, 106.4, 73.4, 64.7, 61.8, 50.1, 30.5, 30.3, 24.3. LCMS (Method-C): RT= 2.25 min; [M+H]^+^ 420.4HPLC (UV, λ_220_ _nm_): 95.07%. HRMS (ESI+) calc’d for C23H25N5O3 [M+H]^+^: 420.2036, found 420.2045

**Synthesis of 3-(4-((7-azaspiro[3.5]nonan-2-yl)oxy)-2H-benzo[d][1,2,3]triazol-2-yl)piperidine-2,6-dione (Cpd-10), 3-(4-((7-benzyl-7-azaspiro[3.5]nonan-2-yl)oxy)-2H-benzo[d][1,2,3]triazol-2-yl)piperidine-2,6-dione (11) & 3-(4-((7-benzoyl-7-azaspiro[3.5]nonan-2-yl)oxy)-2H-benzo[d][1,2,3]triazol-2-yl)piperidine-2,6-dione (12):**

**Step-1: *tert*-butyl 2-(3-amino-2-nitrophenoxy)-7-azaspiro [3.5] nonane-7-carboxylate (III):**

To an ice cold solution of *tert*-butyl 2-hydroxy-7-azaspiro[3.5]nonane-7-carboxylate (**I,** 464 mg, 1.9 mmol) in THF (10 mL) was added NaH (60% suspension in mineral oil; 114 mg) and stirred for 30 min. 3-fluoro-2-nitroaniline (**II,** 300 mg, 1.9 mmol) was added and the resulting reaction mixture was stirred for 16h. After completion, the reaction mixture was quenched with saturated solution of NH_4_Cl and the reaction mixture was diluted with ethyl acetate (10 mL) and washed with water (10 mL). The aqueous layer was back extracted with ethyl acetate (10 mL) and the combined organic layer was washed with water (10 mL), brine, dried over anhydrous Na_2_SO_4_, filtered. The filtrate was concentrated under reduced pressure and the crude material was purified by Combi flash using EtOAc-Hexane (1:5) as an eluent to afford *tert*-butyl 2-(3-amino-2-nitrophenoxy)-7-azaspiro[3.5]nonane-7-carboxylate (**III,** 679 mg, 94%) as a yellow solid, which was used directly to next step without further purification. ^1^H NMR (400 MHz, DMSO) δ 7.09 (t, J = 8.2 Hz, 1H), 6.42 (d, *J* = 8.4 Hz, 1H), 6.12 (d, *J* = 8.1 Hz, 1H), 5.99 (s, 2H), 4.81 – 4.73 (m, 1H), 3.30 – 3.18 (m, 4H), 2.41 – 2.32 (m, 2H), 1.80 – 1.71 (m, 2H), 1.53 – 1.45 (m, 4H), 1.38 (s, 9H). LCMS (Method A): RT= 2.33 min; [M+H] ^+^ 378.3

**Step-2: *tert*-butyl 2-(2,3-diaminophenoxy)-7-azaspiro [3.5] nonane-7-carboxylate (IV):**

To a stirred solution of the *tert*-butyl 2-(3-amino-2-nitrophenoxy)-7-azaspiro [3.5] nonane-7-carboxylate (**III,** 600 mg, 1.62 mmol) in methanol (20 mL) was added HCOONH_4_ (500 mg, 7.92 mmol) followed by the addition of Zn dust (516.7 mg, 7.92 mmol) and the reaction mixture was stirred for 3 h. After completion the reaction mixture was filtered through celite bed. The filtrate was concentrated under reduced pressure. The crude was partitioned between H_2_O (20 mL) and EtOAc (30 mL). The organic layer was separated, dried over Na_2_SO_4_, filtered and the filtrate was concentrated to afford *tert*-butyl 2-(2,3-diaminophenoxy)-7-azaspiro [3.5] nonane-7-carboxylate (**IV,** 521 mg, 93%) which was used directly in the next step without further purification. ^1^H NMR (400 MHz, DMSO) δ 6.36 – 6.27 (m, 1H), 6.19 (d, *J* = 7.8 Hz, 1H), 6.03 (d, *J* = 8.0 Hz, 1H), 4.68 – 4.57 (m, 1H), 4.44 (s, 2H), 3.96 (s, 2H), 3.31 – 3.18 (m, 4H), 2.39 – 2.29 (m, 2H), 1.85 – 1.76 (m, 2H), 1.54 – 1.45 (m, 4H), 1.41-1.36 (m, 9H). LCMS (Method A): RT= 1.86 min; [M+H] ^+^ 348.3

**Step-3: *tert*-butyl 2-((2H-benzo[d] [1,2,3] triazol-4-yl) oxy)-7-azaspiro [3.5] nonane-7-carboxylate (V):**

At -10 °C to a solution of *tert*-butyl 2-(2,3-diaminophenoxy)-7-azaspiro [3.5] nonane-7-carboxylate (**IV,** 521 mg, 1.5 mmol) in glacial acetic acid (30 mL) was added NaNO_2_ (104 mg, 1.5 mmol) as a solution in water (30 mL) and the reaction mixture was stirred for 30 min at ambient temperature. The reaction mixture was then filtered through a pad of celite. The filtrate was concentrated to dryness, dissolved in ethyl acetate (30 mL) and washed with water (30 mL), brine, dried over anhydrous Na_2_SO_4_ and concentrated under reduced pressure to obtain the crude which was purified by silica gel column chromatography using EtOAc-hexane (1:4) as an eluent to provide *tert*-butyl 2-((2H-benzo[d][1,2,3]triazol-4-yl)oxy)-7-azaspiro[3.5]nonane-7-carboxylate (**V,** 358.0 mg, 67%) as a yellow solid.

^1^H NMR (400 MHz, MeOD) δ 7.61 – 7.14 (m, 2H), 6.87 – 6.52 (m, 1H), 5.14 – 4.95 (m, 1H), 3.48 – 3.36 (m, 4H), 2.63 – 2.53 (m, 2H), 2.13 – 2.04 (m, 2H), 1.71 – 1.61 (m, 4H), 1.44 (s, 9H). LCMS (Method A): RT= 2.02 min; [M+H] ^+^ 359.3

**Step-4: *tert*-butyl 2-((2-(2,6-dioxopiperidin-3-yl)-2H-benzo[d] [1,2,3] triazol-4-yl) oxy)-7-azaspiro [3.5] nonane-7-carboxylate (VII):**

To an ice cold solution of NaH (60 wt% in mineral oil, 60 mg, 1.5 mmol) in THF (15 mL) was added *tert*-butyl 2-(2H-1,2,3-benzotriazol-4-yloxy)-7-azaspiro[3.5]nonane-7-carboxylate (**V,** 358 mg, 1.0 mmol) and the mixture was stirred for 20 min, after which this solution was added to the 3-bromopiperidine-2,6-dione (**VI,** 191.78 mg, 1.0 mmol) in THF (5.0 mL) at 0 °C. The reaction mixture was stirred at rt for 6 h. The reaction mixture was poured into 50% saturated aqueous NH_4_Cl. The organic layer was separated, and the aqueous layer was further extracted with EtOAc (2 x10 mL). The combined organic part was washed with brine, dried over anhydrous Na_2_SO_4_, filtered and the filtrate was concentrated. The resulting crude material was purified by silica gel column chromatography (20-100% EtOAc/Hexane) to provide *tert*-butyl 2-((2-(2,6-dioxopiperidin-3-yl)-2H-benzo[d][1,2,3] triazol-4-yl) oxy)-7-azaspiro[3.5]nonane-7-carboxylate (**VII,** 376 mg, 80%) as white solid. ^1^H NMR (400 MHz, DMSO) δ 11.47 – 11.05 (m, 1H), 7.44 (d, *J* = 8.6 Hz, 1H), 7.37 – 7.29 (m, 1H), 6.66 (d, *J* = 7.4 Hz, 1H), 6.17 – 6.08 (m, 1H), 5.03 – 4.92 (m, 1H), 3.34 – 3.27 (m, 2H), 3.25 – 3.19 (m, 2H), 2.95 – 2.68 (m, 3H), 2.59 – 2.52 (m, 1H), 2.49 – 2.42 (m, 2H), 1.98 – 1.87 (m, 2H), 1.61 – 1.48 (m, 4H), 1.39 (s, 9H). LCMS (Method D): RT= 6.39 min; [M-H]^-^ 468.2

**Step-5: Synthesis of 3-(4-((7-azaspiro [3.5] nonan-2-yl) oxy)-2H-benzo[d][1,2,3] triazol-2-yl) piperidine-2,6-dione hydrochloride (10):**

To an ice cold solution of	*tert*-butyl 2-{[2-(2,6-dioxopiperidin-3-yl)-2H-1,2,3-benzotriazol-4-yl]oxy}-7-azaspiro[3.5]nonane-7-carboxylate (**VII,** 100 mg, 0.2 mmol) in DCM (10 mL)	was added HCl in ether (2M; 2.0 mmol) and the resulting reaction mixture was stirred for 2 h. The solvent was removed under vacuo and the crude material triturated with diethyl ether and pentane to obtain 3-(4-((7-azaspiro[3.5]nonan-2-yl)oxy)-2H-benzo[d][1,2,3]triazol-2-yl)piperidine-2,6-dione hydrochloride (**10,** 70 mg, 80%) as an off white solid which was used for next step without further purification. ^1^H NMR (401 MHz, DMSO) δ 11.30 (s, 1H), 8.54 (br s, 2H), 7.45 (d, *J* = 8.6 Hz, 1H), 7.33 (t, J = 7.9 Hz, 1H), 6.66 (d, *J* = 7.5 Hz, 1H), 6.13 (dd, *J* = 12.0, 5.0 Hz, 1H), 5.01 – 4.93 (m, 1H), 3.12 – 2.94 (m, 4H), 2.94 – 2.68 (m, 3H), 2.62 – 2.52 (m, 2H), 2.49 – 2.38 (m, 1H), 2.05 – 1.95 (m, 2H), 1.85 – 1.73 (m, 4H). ^13^C NMR (100 MHz, DMSO) δ 172.4, 168.9, 147.5, 145.4, 136.9, 127.6, 115.6, 109.6, 105.4, 67.9, 64.7, 34.8 (2C), 32.4 (2C), 30.3 (2C), 30.1, 24.3. LCMS (Method C): RT= 2.12 min; [M+H]^+^ 370.3. HRMS (ESI+) calc’d for C19H23N5O3 [M+H]^+^: 370.1879, found 370.1884

**Step-6: Synthesis of 3-(4-((7-benzoyl-7-azaspiro [3.5] nonan-2-yl) oxy)-2H-benzo[d][1,2,3] triazol-2-yl) piperidine-2,6-dione** **(12):**

To a well stirred solution of 3-(4-{7-azaspiro[3.5]nonan-2-yloxy}-2H-1,2,3-benzotriazol-2-yl)piperidine-2,6-dione hydrochloride (**10,** 15 mg, 0.04 mmol) in DCM (10 mL)was added Et_3_N (0.01 mL, 0.07 mmol), benzoyl chloride (**VIII,** 5.71 mg, 0.04 mmol) and stirred at room temperature for 2h.The reaction was monitored by LCMS. Reaction mass was washed with water, organic part dried over anhydrous sodium sulphate. The crude was purified by combi flash column chromatography to afford the3-[4-({7-benzoyl-7-zaspiro [3.5] nonan-2-yl} oxy)-2H-1,2,3-benzotriazol-2-yl] piperidine-2,6-dione (**12,** 15 mg, 78%) as white solid.

^1^H NMR (400 MHz, DMSO) δ 11.27 (s, 1H), 7.43 - 7.30 (m, 6H), 6.67 (d, *J* = 6.8 Hz, 1H), 6.62 (dd, *J* = 12,2, 4.8 Hz, 1H), 4.99 (br s, 1H), 3.61-3.50 (m, 2H), 3.32-3.21 (m, 1H), 3.05 (brs, 2H), 2.90 – 2.66 (m, 3H), 2.58 – 2.54 (m, 1H), 2.49 – 2.38 (m, 2H), 2.04 – 2.01 (m, 2H), 1.69 – 1.64 (m, 4H), 1.20 (br s, 1H). ^13^C NMR (100 MHz, DMSO) δ 172.3, 168.9, 168.8, 147.6, 145.4, 136.9, 136.3, 129.2, 128.3, 127.6, 126.5, 109.5, 105.4, 68.2, 64.7, 45.4, 31.7, 30.3, 24.3. LCMS (Method C): RT= 2.51 min; [M+H]^+^ 474.4. HPLC (UV, λ_220_ _nm_): 98.04%. HRMS (ESI+) calc’d for C26H27N5O4 [M+H]^+^: 474.2141, found 474.2157

**Step-7: Synthesis of 3-(4-((7-benzyl-7-azaspiro [3.5] nonan-2-yl) oxy)-2H-benzo[d][1,2,3] triazol-2-yl) piperidine-2,6-dione (11):**

To a stirred solution of 2-{[2-(2,6-dioxopiperidin-3-yl)-2H-1,2,3-benzotriazol-4-yl]oxy}-7-azaspiro[3.5] nonan-7-ium (**10,** 100 mg, 0.27 mmol) in MeOH (5 mL) was added  benzaldehyde (**IX,** 0.036 mL, 0.41 mmol) followed by the addition of  Sodium cyanoborohydride (33.9 mg, 0.54 mmol) at 0^o^C and the reaction mixture was further stirred at room temperature for 16h. The crude then evaporated under reduce pressure at low temperature and purified by reverse phase prep HPLC to get 3-[4-({7-benzyl-7-azaspiro [3.5] nonan-2-yl} oxy)-2H-1,2,3-benzotriazol-2-yl] piperidine-2,6-dione (**11,** 24.18 mg, 19.4%).

^1^H NMR (401 MHz, DMSO) δ 7.43 (d, J = 8.4 Hz, 1H), 7.34 - 7.23 (m, 5H), 6.64 (d, *J* = 7.6 Hz, 1H), 6.13-6.10 (m, 1H), 4.94 (t, J = 7.0 Hz, 1H), 3.42 (s, 2H), 2.89 - 2.84 (m, 1H), 2.73 - 2.67 (m, 1H), 2.50-2.47 (m, 2H), 2.33 (br s, 1H), 2.26 (br s, 2H), 1.90 (br s, 5H), 1.63 – 1.59 (m, 3H). ^13^C NMR (100 MHz, DMSO) δ 172.3, 168.9, 147.6, 145.4, 138.5, 137.0, 128.7, 128.0, 127.7, 126.7, 109.4, 105.4, 68.4, 64.7, 62.3, 50.0, 49.7, 36.3, 31.2, 30.3, 24.3. LCMS (Method F): RT= 2.44 min; [M+H]^+^ 460.4. HPLC (UV, λ_220_ _nm_): 95.49%. HRMS (ESI+) calc’d for C26H29N5O3 [M+H]^+^: 460.2349, found 460.2356

**Synthesis of 3-(4-((4-(morpholinomethyl)benzyl)oxy)-2H-benzo[d][1,2,3]triazol-2-yl)piperidine-2,6-dione (13):**

**Step-1: Synthesis of methyl 4-[(morpholin-4-yl) methyl] benzoate (III):**

To a well stirred solution of methyl 4-(bromomethyl) benzoate (**II,** 2.0 g, 8.73 mmol) in ACN (20 mL)   morpholine (**I,** 0.76 g, 8.73 mmol), KI (catalytic) and K_2_CO_3_ (3.01 g, 21.83 mmol) were added and stirred at rt for 16h. The reaction was monitored by the LCMS. Reaction mixture was concentrated under reduced pressure to get the crude product which was purified by combi flash column chromatography to afford methyl 4-[(morpholin-4-yl) methyl] benzoate (**III,** 2.0 g, 97.36%) as sticky solid.

^1^H NMR (400 MHz, DMSO) δ 7.92 (d, *J* = 8.2 Hz, 2H), 7.46 (d, *J* = 8.1 Hz, 2H), 3.84 (s, 3H), 3.58 - 3.56 (m, 4H), 3.53 (s, 2H), 2.35 (br s, 4H). LCMS (Method-E): RT= 1.64 min; [M+H]^+^ 236.2

**Step-2: Synthesis of {4-[(morpholin-4-yl) methyl] phenyl} methanol (IV):**

To a stirred solution of methyl 4-[(morpholin-4-yl) methyl] benzoate (**III**, 600 mg, 2.55 mmol) in THF (6.0 mL) was added LAH (2M in THF, 3.8 mL) at 0°C and stirred at rt for 10 mins.  and further continued for 2h. After completion the reaction, saturated aqueous Na_2_SO_4_ solution was added dropwise at 0 °C. Then it was filtered off and concentrated under reduced pressure to get the crude product which was purified by combi flash column chromatography to afford the {4-[(morpholin-4-yl) methyl] phenyl} methanol (**IV,** 505 mg, 95%) as sticky solid. ^1^H NMR (400 MHz, DMSO) δ 7.27 - 7.23 (m, 4H), 5.12 (t, *J* = 5.7 Hz, 1H), 4.46 (d, *J* = 5.7 Hz, 1H), 3.56 - 3.54 (m, 4H), 3.42 (s, 2H), 2.32 (brs, 4H). LCMS (Method-E): RT= 1.33 min; [M+H]^+^ 208.2

**Step-3: Synthesis of 3-((4-(morpholino methyl) benzyl) oxy)-2-nitroaniline (VI):**

To a stirred solution of {4-[(morpholin-4-yl) methyl] phenyl} methanol (**IV,** 500.0 mg, 2.41 mmol) in THF (8.0 mL) was added NaH (60% in oil, 250 mg, 6.03 mmol) at 0°C and stirred at rt for 10 min.   3-fluoro-2-nitroaniline (**V,** 376.6 mg, 2.41 mmol) was added and continued to stir at RT for 16h.The reaction was monitored by the LCMS. Reaction mixture was concentrated under reduced pressure to get the crude product. The crude was purified by combi flash column chromatography to afford 3-((4-(morpholino methyl) benzyl) oxy)-2-nitroaniline (**VI,** 500 mg, 60%) as off white solid.

^1^H NMR (400 MHz, DMSO) δ 7.37 - 7.31 (m, 4H), 7.13 (t, *J* = 8.3, Hz, 1H), 6.46 (d, *J* = 8.3 Hz, 1H), 6.39 (d, *J* = 8.3 Hz, 1H), 6.02 (brs, 2H), 5.12 (s, 2H), 3.57 - 3.46 (m, 4H), 3.45 (s, 2H), 2.34 - 2.33 (m, 2H). LCMS (Method-E): RT= 1.78 min; [M+H]^+^ 344.2

**Step-4: Synthesis of 3-((4-(morpholino methyl) benzyl) oxy) benzene-1,2-diamine (VII):**

To a stirred solution of 3-({4-[(morpholin-4-yl) methyl] phenyl} methoxy)-2-nitroaniline (**VI,** 700 mg, 2.04 mmol) in MeOH (10 mL) were added Ammonium formate (642.8 mg, 10.19 mmol) and Zn powder (666.5 mg, 10.19 mmol) into the mixture and continued to stir at room temperature for 12h. The reaction was monitored by the LCMS. Reaction mixture was concentrated under reduced pressure to get the crude product. The crude was purified by combi flash column chromatography to afford 3-((4-(morpholino methyl) benzyl) oxy) benzene-1,2-diamine (**VII,** 577 mg, 90%).

^1^H NMR (400 MHz, DMSO) δ 8.27 (s, 4H), 7.41 (d, *J* = 7.6 Hz, 2H), 7.30 (d, *J* = 7.6 Hz, 2H), 6.37 - 6.33 (m, 1H), 6.28 (d, *J* = 8.0 Hz, 1H), 6.22 (d, J = 7.7 Hz, 1H), 5.00 (s, 2H), 3.56 (s, 4H), 3.43 (s, 2H), 2.34 (m, 4H). LCMS (Method-E): RT= 1.59 min; [M+H]^+^ 314.3

**Step-5: Synthesis of 4-(4-(((2H-benzo[d][1,2,3] triazol-4-yl) oxy) methyl) benzyl) morpholine (VIII):**

To a well stirred solution of 3-({4-[(morpholin-4-yl) methyl] phenyl} methoxy) benzene-1,2-diamine (**VII,** 577 mg, 1.84 mmol) in AcOH (5 mL)    H_2_O (5 mL)    was added NaNO_2_ (139.88 mg, 2.02 mmol) at 0°C and stir at rt for 2 h. The reaction was monitored by the LCMS. Reaction mixture was concentrated under reduced pressure to get the crude product. The crude was purified by combi-flash column chromatography to afford 4-(4-(((2H-benzo[d][1,2,3] triazol-4-yl) oxy) methyl) benzyl) morpholine (**VIII,** 400 mg, 67%). ^1^H NMR (400 MHz, DMSO) δ 15.80 (brs, 1H), 7.51 - 7.49 (m, 3H), 7.37 - 7.35 (m, 3H), 6.95 (br s, 1H), 5.35 (s, 2H), 3.58 - 3.56 (m, 4H), 3.47 (s, 2H), 2.36-2.33 (m, 4H). LCMS (Method-E): RT= 1.59 min; [M+H]^+^ 325.3

**Step-6: Synthesis of 3-[4-({4-[(morpholin-4-yl) methyl] phenyl} methoxy)-2H-1,2,3-benzotriazol-2-yl] piperidine-2,6-dione (13):**

To a well stirred solution of 4-({4-[(morpholin-4-yl) methyl] phenyl} methoxy)-2H-1,2,3-benzotriazole (**VIII**, 100 mg, 0.31 mmol) in THF (3 mL) was added NaH (60%, 31 mg, 0.77 mmol) at 0°C and stirred at rt for 10 min.   3-bromopiperidine-2,6-dione (**IX,** 147.98 mg, 0.77 mmol) was added and continued to stir at RT for 16h.The reaction was monitored by the LCMS. The reaction mixture was quenched with cold water and extracted by EtOAc. Organic layer was separated, dried over Na_2_SO_4_ and concentrated under reduced pressure to get the crude product. The crude was purified by combi-flash column chromatography to afford the desired compound 3-[4-({4-[(morpholin-4-yl) methyl] phenyl} methoxy)-2H-1,2,3-benzotriazol-2-yl] piperidine-2,6-dione (**13,** 45 mg, 33.52 %).

^1^H NMR (400 MHz, DMSO) δ 11.26 (s, 1H), 7.49 - 7.47 (m, 3H), 7.39 - 7.35 (m, 3H), 6.93 (d, *J* = 7.5 Hz, 1H), 6.15 - 6.12 (m, 2H), 5.29 (s, 2H), 3.57 (br s, 4H), 3.47 (s, 2H), 2.90 - 2.81 (m, 2H), 2.72 - 2.67 (m, 2H), 2.12 (br s, 4H). ^13^C NMR (100 MHz, DMSO) δ 172.3, 168.9, 149.0, 145.4, 137.8, 137.0, 135.0, 129.0 (2C), 128.0 (2C), 127.6, 109.7, 105.4, 69.8, 66.1 (2C), 64.7, 62.1, 53.1 (2C), 30.3, 24.3. LCMS (Method-F): RT= 2.39 min; [M+H]^+^ 436.3**.** HPLC (UV, λ_220_ _nm_): 98.34%. HRMS (ESI+) calc’d for C23H25N5O4 [M+H]^+^: 436.1985, found 436.1992

**CRBN-DDB1 binding assay**

The fluorescent probe Cy5-labeled Thalidomide (Tocris, cat. 7288) capable of binding CRBN was used in the binding assay. Compounds with 100 nM DDB1-CRBN (R&D Systems, cat. E3-500) were preincubated for 15 minutes prior to the addition of 20 nM Cy5 probe and further incubated for 30 minutes at room temperature. Interaction between compound and protein was assessed by changes in the fluorescence polarization of Cy5 probe. IC_50_ values were calculated with respect to vehicle control (DMSO) with and without DDB1-CRBN protein.

**Ikaros Degradation Assay**

SU-DHL-10 was cultured RPMI in media with 10% heat-inactivated serum. For testing the compounds, 0.5 x 106 cells were plated along with the respective dose of test compounds at 0.1% DMSO and incubated at 37°C, 5% CO2. After 24 h of compounds treatment, cells were harvested, washed with 1X PBS and stored at -80°C for further analysis. On the day of Western Blot analysis, the cell pellets were thawed on ice and lysed with buffer containing 0.1% TritonX-100 and Halt^TM^ protease inhibitor cocktail. The total protein content for each sample was measured using BCA method (Sigma# QPBCA-1KT) and diluted to 0.75 µg/µl for analysis. Samples for detection of IKAROS in the treated and control (vehicle) cell lysates were prepared following protocol for Simple Western^TM^ method. IKAROS protein in the cell lysate were detected using anti-IKAROS antibody (CST#5443S). The total amount of target protein was normalized by the GAPDH protein (detected using antibody, CST# 5174S) in respective sample and treated sample were compared with that of vehicle samples.

**Cell viability assay**

HEK293 cells were cultured in complete growth media consisting of DMEM (Gibco, cat. 11960-044) supplemented with 10% fetal bovine serum (Gibco, cat. 10082147) and 1% penicillin/streptomycin (Gibco, cat. 15140122). Cells were seeded at 1000 cells/well in 384-well plate (Greiner bio-one, cat. 781080) in complete growth media and incubated overnight at 37°C, 5% CO_2_. The following day, compounds/vehicle control (DMSO) were added to the cell plate at 10X concentration at 5% DMSO (final assay concentration of DMSO: 0.5%) and incubated for 72 hours at 37°C, 5% CO_2_. CellTiter-Glo® reagent (Promega, cat. G7572) was added to the wells and plate contents were mixed on an orbital shaker for 2 minutes then incubated for 15 minutes at room temperature. The luminescence signal was detected using EnVision (PerkinElmer) multimode reader. Cell viability was calculated as percentage of vehicle control.

**Docking study**

5V3O, the co-crystal structure of the human cereblon (chain C) in complex with DDB1 (DNA damage binding protein 1, chain A) has been disclosed (Matyskiela et al. 2018) and was used for our docking studies which was downloaded from RCSB PDB database. C-chain from the PDB structure, representing the cereblon, was extracted along with the bound ligand, and then hydrogen-assigned and energy minimized in Accelrys Discovery Studio visualizer. This was done to accommodate the changes induced by the bound ligand on the binding site. The residues in the resulting PDB file were assigned partial atomic charges according to CHARMM using in-house algorithm (FORTRAN code).

Self-ligand was extracted from the PDB, hydrogen-assigned and energy minimized in Accelrys Discovery studio visualizer. All the other small molecules were drawn in 2D in ChemDrawUltra, then exported as mol files and opened in Accelrsys Discovery Studio visualizer to assign hydrogen and energy minimized and saved as PDB files and mol files simultaneously. The self-ligand and other compound files were then subjected to a series of in-house algorithms (both PERL scripts & FORTRAN codes) to calculate their rotatable bond information and assign partial atomic charges.

X-Y-Z coordinate (44.215 53.752 -38.073), a position near the center of the bound ligand in 5V3O was taken as the center of the binding site for docking of the self-ligand, compound 13-*S* & 13-*R* as they are similar in size. For compounds 1 & 4, which are much smaller in dimension, the binding site center was chosen as (X-Y-Z: 46.289 53.155 -38.382) which was near the center of the thalidomide moiety of the bound ligand. Each molecule was then subjected to docking by in-house algorithm (FORTRAN code), which placed the compound in the binding site at the center and used 100000 iterations to generate random conformers on the fly and calculated binding energies. Energy cut-off was kept at 0 kcal/mol. Conformers for which ligand atoms were closer to 1Å to the protein atoms were considered too close, thus contributing to steric hindrance, and hence discarded; and for conformers with atoms at 18Å or further from the protein atoms were considered too far away to make significant contribution to binding energies. The pose with the lowest energy was considered as the best docked pose.

**^1^H, ^13^C NMR & HRMS of Cpd-4:**

**n*O*e data of 4:**

No n*O*e was observed between NCH proton H13 and phenyl ring doublet proton H1 / H4 which supports the formation of desired structure.

**^1^H, ^13^C NMR & HRMS of Cpd-5:**

**^1^H, ^13^C NMR & HRMS of Cpd-6:**

**^1^H, ^13^C NMR & HRMS of Cpd-7:**

**^1^H, ^13^C NMR & HRMS of Cpd-8:**

**^1^H, ^13^C NMR & HRMS of Cpd-9:**

**^1^H, ^13^C NMR & HRMS of Cpd-10:**

**^1^H, ^13^C NMR & HRMS of Cpd-11:**

**^1^H, ^13^C NMR & HRMS of Cpd-12:**

**n*O*e data of Cpd-12:**

**^1^H, ^13^C NMR & HRMS of Cpd-13:**

**n*O*e data of Cpd-13:**

No n*O*e was observed between NCH proton H32 and H3 / H10 which supports the formation of desired structure.
